# Joeropsididae Nordenstam, 1933 (Crustacea, Isopoda, Asellota) from the Lizard Island region of the Great Barrier Reef, Queensland, Australia

**DOI:** 10.3897/zookeys.491.4932

**Published:** 2015-03-26

**Authors:** Niel L. Bruce

**Affiliations:** 1 Museum of Tropical Queensland, Queensland Museum, 70–102 Flinders Street, Townsville, Australia 4810; 2 Water Research Group (Ecology), Unit for Environmental Sciences and Management, North West University, Potchefstroom, 2520, 2006 South Africa; 3 College of Marine and Environmental Sciences, James Cook University, Townsville, Queensland, Australia

**Keywords:** Isopoda, Asellota, *Joeropsis*, coral reef, Australia, southwestern Pacific, taxonomy

## Abstract

The marine isopod family Joeropsididae (Asellota) is documented for the Lizard Island region of the Great Barrier Reef, Australia. Fifteen species of *Joeropsis* are recorded, including ten new species; descriptive notes are provided for five species that lacked adequate material for description. A revised family and genus diagnosis is presented together with comments on the most useful characters for species identification and a key to *Joeropsis* of the Lizard Island region.

## Introduction

The family Joeropsididae Nordenstam, 1933 and the genus *Joeropsis* Koehler, 1885 both have a global distribution, being absent only from polar waters (Schotte et al. 2011). *Joeropsis* is well represented in tropical regions worldwide, with more than 58% (53 of 86, including undescribed Australian species) of the known species from shallow (< 50 metres) coastal waters and coral reefs. Of the known species, only seven have been reported from depths greater than 300 metres, with *Joeropsis antarctica* Menzies & Schultz, 1968 recorded from 1,408 metres off the South Shetland Islands, Antarctica.

*Joeropsis* appears to be ubiquitous in coral-reef habitats with previous records indicating diversity in the order of two to six species in any region or locality. For example six species are known from coral reefs of the Seychelles and its territories (Kensley and Schotte 2002), two species from the Mascarene Islands (Müller 1991a), three species from the Society Islands (Müller 1989) and four species from Easter Island (Kensley 2003). The collecting methods used by those workers did not involve SCUBA so were restricted to the intertidal or very shallow (<2 metres) depths, or were from ship-based dredges or sleds. Collection methods used during the Census of Marine Life CReefs (see http://www.aims.gov.au/creefs) Lizard Island expeditions obtained a far higher number of both specimens and species, indicating the species diversity for this genus on Australian coral reefs may be in the order of 10 to 15 species per region to approximately 30 metres depth.

Knowledge of the Australian fauna rests with the single contribution of Just (2001), later summarised by Poore and Lew Ton (2002), totalling three species in three genera, all from the Bass Strait and Tasmania, south-eastern Australia. A fourth species was described from Macquarie Island, Australia Territory in the Southern Ocean (Hale 1937). No species of Joeropsididae had been recorded from tropical waters anywhere in Australia prior to this work.

## Material and methods

**Sampling.** Shallow coral-reef habitats can be broadly divided into two convenient categories: inter-tidal reef flat and sub-tidal outer reef to about 30 metres. Algae can be regarded as a sub-category of both. Both categories are sampled in much the same way. Samples of dead coral substrate (including fossil or compacted reef, eroded and dead coral heads; coral rubble was particularly productive) were collected by hand into a 20–25 litre plastic bucket and moderately broken up in the laboratory, the water laced with a few drops of concentrated formaldehyde and left to stand for 5–30 minutes. Small samples were collected in 250 μm or 350 μm mesh bags and processed the same way. The sample was then elutriated (= rinsed) using a seawater hose with the washings passed through a wet sieve or fine-mesh net and either sorted immediately under a microscope or fixed in formaldehyde or ethanol for later sorting. Other methods included ethanol rinsing and freshwater rinsing of samples.

Sand samples were collected by gently scraping and excavating by hand into a ‘ziplok’ plastic bag, usually taking a volume of less than one litre, then formalin rinsed in a tray and sieved through a net. Mobile sand at the base of gullies or bommies^1^, and sand accumulation on top of bommies or ridges were particularly productive.

These techniques were used for sampling both intertidal reef flats and subtidal reef slopes to 30 metres, by snorkelling or using SCUBA. Shallow (<2 metres) subtidal sand samples were only taken by SCUBA, being impractical to sample while snorkelling.

**Figure 1.**
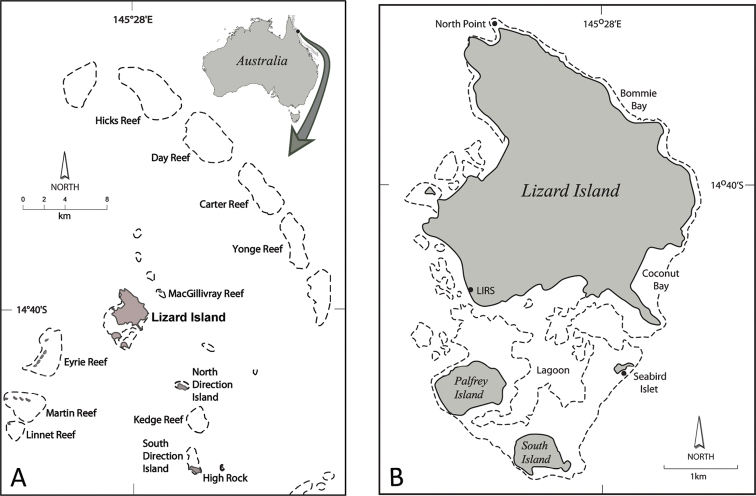
CReefs collecting sites in the Lizard Island region with names of major collecting sites. **A** Lizard Island group and nearby islands and reefs **B** Lizard Island group. LIRS = Lizard Island Research Station.

Principal sites are shown in Figure 1. Sampling was carried out under GBRMPA Permit G08–27858.1 and General Fisheries Permit (QLD DPI) 95152.

**Descriptions.** Conventions largely follow Bruce (2009a, 2009b) with some minor changes in terminology. The dactylar ungui of Asellota are traditionally referred to as ‘claws’ and that term is used here. Pleopods 4 and 5 are simple lobes and never feature in species identification or in the characterisation of higher taxa; furthermore they are tiny and difficult to extract and are routinely not figured. Pereopods 1–7 are closely similar and only pereopods 1, 2 and 7, or 1 and 7 are illustrated. Pleopod 1 has the rami fused—width measurements refer to the paired rami. Width in all cases refers to maximum width, unless otherwise specified. All species descriptions are based on the holotype.

**Dissections.** CReefs samples were all preserved in high-grade ethanol (a requirement of the CReefs program), without first fixing in formalin. Such material is initially brittle and over time remains very fragile. Dissections from a single specimen, particularly of small species (<2 mm) resulted in effective destruction of the specimen and dissected appendages are often broken. Consequently, descriptions given here may be from several specimens, and for those species represented by few specimens dissection was strictly minimised. Species represented by single specimens or few damaged specimens have not been described, but have been included in order to document overall species diversity in the region of Lizard Island. Microslide preparations were made using CMCP-9 or Lactic Acid stained with lignin pink, the appendages being remounted in Gurr’s Aquamount; or whole animals stained and partly cleared in CMCP-9 and then dissected and mounted in Aquamount. Transferring minute mounted appendages from Lactic Acid to Aquamount resulted in some losses of appendages. Some dissections failed.

**Names.** Classical names were derived using Brown (1956) except where otherwise stated; Aboriginal names were taken from Anonymous (1965).

**Abbreviations**

LIRS Lizard Island Research Station;

MTQ Museum of Tropical Queensland, Townsville;

QM Queensland Museum, Brisbane;

SMF Senckenberg Institute, Frankfurt;

RS robust seta/e.

**Primary collectors**

NLB Niel L. Bruce;

MB-P Magda Błażewicz-Paszkowycz (University of Lodz, Poland);

CB Chad Buxton;

JC Julian Caley (Australian Institute of Marine Science, Townsville).

## Systematics

### Family Joeropsididae

Taxon classificationAnimaliaIsopodaJoeropsididae

Nordenstam, 1933

Jaeropsinae Nordenstam, 1933: 190.
Iaeropsinae . – Nierstrasz 1941: 288.
Jaeropsidae . – Menzies 1962: 63. – Menzies and Glynn 1968: 75.
Joeropsididae . – Sivertsen and Holthuis 1980: 96. – Wilson 1997: 86. – Kussakin 1999: 10. – Just 2001: 303.

#### Diagnosis.

**Male.** Body dorsoventrally flattened; lateral margins normally parallel, occasionally tapering posteriorly. Pereonites of subequal length; lateral margins covering coxae in dorsal view, entire, smooth or finely serrate. Pereopods all ambulatory, all similar; pereopod 1 with 2 dactylar claws, pereopods 2–7 with 2 or 3 dactylar claws. Eyes dorsolateral, sessile. Anterior margin of cephalon with strong median concavity. Pseudorostrum present, inserted into cephalic concavity, rarely joined along straight line. Pleon with no free pleonites. Pleotelson subequal in width to pereonite 7. Antenna 1 shorter than cephalon, peduncular article 1 expanded, longer than articles 2 and 3 combined; flagellum shorter than peduncle, with 3–5 articles. Antenna 2 peduncle geniculate, with article 6 and flagellum folding laterally and posteriorly under lateral margin of expanded article 5; first 4 articles short, article 4 more or less embedded into 3, article 5 longer than 1–4 combined, expanded laterally, article 6 0.5–0,8 as long as article 5, generally widening distally; antennal scale absent; flagellum with enlarged, normally conjoint article 1. Mandible molar a long, slender, pointed projection (often with small accessory denticles); incisor of 4–6 large teeth; lacinia mobilis absent; spine row present. Maxilliped sub-quadrate, covering entire mouthpart field, distally margin convex, with distinct distomesial concavity; palp with at least article 2 mesially expanded, epipod half length of endite or less. Pleopod 2 rami with longer or shorter lateral fringe of modified cuticular scales. Pleopod 3 exopod biarticulate, longer than endopod, with lateral fringe of modified cuticular scales; endopod with 3 plumose setae. Pleopod 4 exopod vestigial. Uropods biramous, inserted ventrally on pleotelson usually within distinctive insinuation in pleotelson margin; peduncle broader than long and mesially expanded; rami shorter than peduncle. Anus outside pleopodal chamber, between bases of uropodal peduncles, partly or entirely covered by pleopod 1.

**Female.** Pleopod 2 with lateral fringe of cuticular scales; partly or entirely covering anus. Female spermathecal duct opening on anterior surface of pereonite 5, oviduct opening ventrally on pereonite 5 mesially to coxa. Oostegites on pereopods 1–5.

#### Included genera.

*Joeropsis* Koehler, 1885, *Rugojoeropsis* Just, 2001 and *Scaphojoeropsis* Just, 2001.

#### Remarks.

Only a few species of the family Joeropsididae had been described by the 1990s. By 1950 eleven species of *Joeropsis* had been named, and a further 13 species had been described by 1975. In a period of high activity in the late twentieth century (see Poore and Bruce 2012), notably by Hans-Georg Müller and Brian Kensley, a further 29 species were named (see Schotte et al. 2011), all the while the family remaining with the single genus. In 2001 Jean Just described the first new genera within the Joeropsididae, and at that point there were 69 known species and two subspecies (Just 2001).

The family is readily recognised, including in the field, by the compact body shape, with a characteristically robust and reflexed antenna 2, and small, ventrolaterally inserted uropods with a peduncle that is large in relation to the tiny rami.

The mouthpart morphology suggests that the highly mobile joeropsidids are carnivorous. The mandibles usually possess a five-cusped incisor with acute cusps, completely lacks a lacinia mobilis, has a prominent spine row of simple or finely serrate spines and a blade-like molar process, all characters that are analogous to the mouthparts of the scavenging or predatory isopod family Cirolanidae. These characters are not unique to the family, but the maxilliped morphology with the large endite, small palp and epipod, and mesially excavate distal margin does appear to be unique.

The phylogenetic relationships of the Joeropsididae remain unsettled. Three analyses have included representatives of the family (Wilson 1994; Rapuach et al. 2009; Lins et al. 2012) but none had the Joeropsididae as the primary focus. Wilson (1994), using morphological data, included the Joeropsididae as a potential outgroup for the Janiridae, and found that the Joeropsididae was sister to *Jaera*+*Iais* (Wilson 2004, figs 1 and 2), but those clades lacked supporting apomorphies. In contrast both Raupach et al. (2009) and Lins et al. (2012), using molecular data, found that the Joeropsididae are closer to the Acanthaspidiidae. Raupach et al. (2009) showed the Joeropsididae as sister group to the Acanthaspidiidae (including *Ianthopsis*) and that Joeropsididae+Acanthaspidiidae are sister to *Iarthrippa* (part of Janiridae) (Raupach et al. 2009, fig. 1, strict consensus tree), or sister to *Iarthrippa* (Raupach et al. 2009, fig. 2, 50% majority rule tree), with Joeropsididae+*Iarthrippa* sister to Acanthaspidiidae+*Ianthopsis*. Lins et al. (2012) found that the Joeropsididae is sister group to the Acanthaspidiidae (Fig. 1). These analyses uphold the monophyly of the Joeropsididae, and indicate a close but unresolved relationship to both the Acanthaspidiidae and Janiridae.

#### Key to the genera of Joeropsididae

**Table:** 

1	Body lateral margins straight; maxillipedal palp article 2 only with distomesial lobe, not widest distally	**2**
–	Body lateral margins converging posteriorly; maxillipedal palp articles 2 and 3 widest distally, each with distomesial lobe	***Scaphojoeropsis***
2	Body dorsal surfaces coarsely granular and nodulose	***Rugojoeropsis***
–	Body dorsal surfaces smooth or finely granular, with or without longitudinal carinae	***Joeropsis***

### Genus Joeropsis

Taxon classificationAnimaliaIsopodaJoeropsididae

Koehler, 1885

Joeropsis Koehler, 1885: 7. – Kensley and Schotte 1989: 87; Wilson 1997: 86; Kussakin 1999: 12; Just 2001: 304; Kensley and Schotte 2002: 1428.
Jæropsis . – Richardson 1905: 476; Stebbing 1905: 50.
Jaeropsis . – Vanhöffen 1914: 531(unjustified emendation). – Nordenstam 1933: 191. – Menzies and Barnard 1959: 10; Menzies 1962: 64; Menzies and Glynn 1968: 76.
Iaeropsis . – Nierstrasz 1941: 288 (unjustified emendation).

#### Diagnosis.

Body lateral margins parallel, with or without dorsal sculpture. Cuticle polished, smooth or finely granular. Pseudorostrum with overhanging apex. Upper lip evenly rounded, less than twice as wide as long. Mandible incisor with 5 or 6 strong subequal evenly spaced cusps; spine row setae long, in regular row, with or without lobe on right mandible spine row. Lower lip, lobes longer than wide, distally tapering, pointed. Maxillipeds endite reaching to end of or beyond palp article 3; palp about half length of endite; palp article 3 without mesial lobe, article 4 much longer than article 3. Pereopod 1 with 2 dactylar claws, pereopods 2–7 with 2 or 3 dactylar claws.

**Female.** Pleopod 2 (operculum) with at most a few short simple setae apically.

#### Type species.

*Joeropsis brevicornis* Koehler, 1885, by monotypy. Menzies (1962) incorrectly stated the type species to be *Joeropsis curvicornis* (Nicolet, 1849) as did Menzies and Glynn (1968). The original orthography on the heading page of Koehler (1885) was *Joeropsis*, thereafter *Jœropsis* [œ] not *Jæropsis* [æ], the derivation from *Jaera* notwithstanding; the two spellings can be indistinguishable depending on the font used, but my interpretation is that in some cases the spelling is ambiguous. I follow the first use—*Joeropsis*.

#### Remarks.

Most older diagnoses (e.g. Menzies and Barnard 1959) contain little diagnostic information. Wilson (1997) provided the first restrictive diagnosis. Just (2001) gave the most recent generic diagnosis. A full synonymy was given by Kussakin (1999) for family and genus, though spelling changes were largely ignored, and the synonymy includes two identical spellings.

A number of authors have, over the years, recorded species of *Joeropsis* from widely disparate locations, some commenting on variation, occasionally establishing subspecies. In most such cases the identity of records remote from the type locality or core distribution have to be regarded with caution and scepticism. The records in the literature, particularly earlier than the 1980s, often lack adequate illustrative and descriptive data. Giving just one example, *Joeropsis curvicornis* (Nicolet, 1849) was recorded from Chile (original record), Sri Lanka (Stebbing 1905) and New Zealand (when *Joeropsis neozelanica* Chilton, 1891 was considered a junior synonym) but these records are highly unlikely to be the one species.

Maxilliped palp article 3 in most species lacks a distomesial lobe or process, the exception being *Joeropsis sanctipauli* Kensley, 1989, which has a small distomesial lobe (Kensley 1989, fig. 3H). Most species of *Joeropsis*, including all Australian species (those described here and by Just [2001]) have a distomesial lobe only on maxilliped palp article 2, the exception being *Joeropsis mije* sp. n., which also has a small distolateral lobe on palp article 1 (Fig. 15D, E).

The mandible incisor has five or six distally acute cusps, usually of similar size. Exceptions are *Joeropsis indica* Müller, 1991b and *Joeropsis makrogenys* sp. n., both of which have markedly asymmetric mandibular incisors, with a truncate mesial cusp on the left mandible with the remaining cusps set on a lobe; in *Joeropsis indica* the right mandible cusps are of the usual form, but in *Joeropsis makrogenys* the right mandible proximal or posterior cusp is conspicuously wide and broadly rounded.

#### Species recognition.

Species within a region are most readily identified by their characteristic colour pattern. Colour pattern is consistent, though shade and density of colour may vary, particularly on preservation. Some species will share similar colour patterns, and for old preserved specimens that have lost the colour pattern morphological characters can be used, the most obvious in the first instance being shape of the pseudorostrum. Other characters that are useful include shape of head (lateral margins narrowing anteriorly, concave, straight; serrate or not), body compactness, antenna 1 and antenna 2 (serrate or not; articles lobed or not; relative width of antenna 2 articles 5 and 6); details of the maxilliped (notably the distal margin of the endite and details of the maxilliped palp), pleotelson shape and serrations of the lateral margins; in some cases the male pleopod 1 will separate species but the differences are often subtle. Eyes are always dorsolateral in position, but vary in size and may be marginal or sub-marginal in position. A small number of species show dorsal sculpting in the form of carinae or low nodules, ventral keels may be present and the uropods may be with (most species) or without (few species) a distomesial spine. Supporting characters can be seen in body proportions and uropods. Generally the mandible is similar throughout the genus, but two species, *Joeropsis indica* Müller 1991b and *Joeropsis makrogenys* sp. n. have the proximal cusps on the left mandible incisor set on a lobe, and both species have a comparatively large labrum; additionally the mandibular incisor right proximal cusp is broadly rounded in *Joeropsis makrogenys*. Pereopods and pleopods are generally uniform throughout the genus, although there are differences in pereopod proportions and setation, including the number of dactylar claws on pereopods 2–7 (2 or 3 claws, and 2 claws with a stiff seta).

#### Sexual dimorphism.

Males and females of *Joeropsis* are generally similar, other than for the primary sexual characters. There are some instances of secondary sexual variation, for example the strongly dimorphic antenna 2 in *Joeropsis mije* sp. n. and *Joeropsis minuta* Müller, 1989 (see Müller 1989, fig. 15F, G), while in *Joeropsis panstikta* sp. n. the males have fewer spines on the pleotelson in comparison to the female.

#### Key to the Lizard Island species of *Joeropsis*

This key applies to the named species, 10 of the 15 recorded species in the region. Identifications should be checked against the remarks given for the listed but undescribed species. Inter-reef habitats beyond diving depth are highly likely to have further undescribed species.

**Table:** 

1	Pereonites 5–7 with sub-median dorsal carinae	***Joeropsis tropida* sp. n.**
–	Body without longitudinal dorsal carinae	**2**
2	Body dorsally coloured on all pereonites and pleotelson	**3**
–	Body with dorsal coloured bands on head only or some pereonites or variously patterned with one or more clear pereonites	**4**
3	Body dorsally evenly dark brown, surfaces moderately setose; (pseudorostrum anteriorly rounded)	***Joeropsis adusta* sp. n.**
–	Body dorsally reddish brown, head always darker than rest of body; surfaces smooth; (pseudorostrum anteriorly rounded)	***Joeropsis panstikta* sp. n.**
4	Head only with transverse dark-brown band	**5**
–	Head and some other somites coloured	**7**
5	Pseudorostrum anteriorly concave; maxilliped endite without excavate distomesial angle	***Joeropsis makrogenys* sp. n.**
–	Pseudorostrum not anteriorly concave; maxilliped endite with excavate distomesial angle	**6**
6	Body 4.7 as long as wide; lateral margins of head not strongly serrate; pseudorostrum lateral margins converging, apex narrowly subtruncate; pleotelson lateral margins each with 5 serrations	***Joeropsis wattora* sp. n.**
–	Body 3.1 as long as wide; lateral margins of head strongly serrate; pseudorostrum anteriorly narrowed, narrowly rounded; pleotelson lateral margins each with 8 serrations	***Joeropsis varanus* sp. n.**
7	Pseudorostrum anteriorly acute	**8**
–	Pseudorostrum rounded, angled or excavate (not anteriorly acute)	**9**
8	Body with four transverse dark bands on head and posterior of pereonites 1, 2 and 4; head and pereonites 1–4 lateral margins serrate	***Joeropsis jiigurru* sp. n.**
–	Body with head only with diffuse band; pereonites without distinct bands (male antenna 2 article 5 near circular in outline)	***Joeropsis mije* sp. n.**
9	Head lateral margins anteriorly narrowed; body dorsally with sparse chromatophores, pereonite 5 clear; pseudorostrum anteriorly rounded	***Joeropsis goobita* sp. n.**
–	Head lateral margins sub-parallel; head band short, diffuse, chromatophores present on pereonites 1–4 and 6 and 7, pereonite 5 clear; pereonites 6 and 7 always paler than 1–4; pseudorostrum anteriorly narrowly excavate	***Joeropsis specca* sp. n.**

### Joeropsis adusta sp. n.

Taxon classificationAnimaliaIsopodaJoeropsididae

http://zoobank.org/8960F365-4D4E-4B3F-A2EB-D2D56ED92CF4

Figs 2 , 3 , 4

#### Material.

*Holotype*. ♂ (1.6 mm), ‘High Rock’, east of South Direction Island, 14.82428°S, 145.55270°E, 11 September 2010, clean coral rubble 6.0 m, stn LI10-134C, coll. CB (MTQ W33715).

*Paratypes*. 13 ♂ (1.1–1.6 mm; 3 damaged), 31 ♀ (11 ovig 1.4–1.8; 20 non-ovig. 1.1–1.7mm), same data as holotype (MTQ W33033). ♂ (2.0 mm), ♀ (ovig 2.2 mm), 2 imm. (1.6, 1.2 mm), Seabird Islet, patch reef, in from lagoon entrance, 14.68900°S, 145.46710°E, 11 April 2008, dead coral heads 1.0–2.0 m, stn CGLI-18A, coll. NLB & MB-P (MTQ W13975).

*Additional material*. 3, Bommie Bay, Lizard Island, 14.66127°S, 145.47130°E, 2 September 2010, dead coral, 6.5 m, stn LI10-57D, coll. CB (MTQ W32756). 12, Bommie Bay, Lizard Island, 14.66127°S, 145.47130°E, 2 September 2010, dead coral on bommie, 3 m, stn LI10-057A, coll. CB (MTQ W32743). 1, Bommie Bay, Lizard Island, 14.66127°S, 145.47130°E, 2 September 2010, coral rubble at base of bommie, 6 m, stn LI10-057B, coll. CB (MTQ W32747). 7, Bommie Bay, Lizard Island, 14.66157°S, 145.47160°E, 8 September 2010, dead coral, 8 m, stn LI10-101B, coll. CB (MTQ W32937). 1, Bommie Bay, Lizard Island, 14.66157°S, 145.47160°E, 8 September 2010, dead coral on wall, 10 m, stn LI10-100C, coll. CB (MTQ W32556). 1, High Rock, 14.82553°S, 145.55170°E, 6 September 2010, fine coral rubble, 6 m, stn LI10-091D, coll. CB (MTQ W32903). 2, High Rock, east of South Direction Island, 14.82462°S, 145.5520°E, 6 September 2010, dead *Acropora* plates, 4 m, stn LI10-092B, coll. CB (MTQ W32921). 1, ‘Washing Machine’, northwest Lizard Island, 14.6482°S, 145.4570°E, 2 September 2010, coral rubble at base of reef, 12 m, stn LI10-056C, coll. CB (MTQ W32730). 9, Yonge Reef, 14.57735°S, 145.61050°E, 10 September 2010, lee side (western) of reef, coral rubble, 10 m, stn LI10-127A, coll. CB (MTQ W32992). 2, Yonge Reef, 14.57735°S, 145.61050°E, 10 September 2010, lee side (western) of reef, coarse coral rubble, 25 m, stn LI10-127F, coll. CB (MTQ W31809). ♀ (non-ovig. 1.3 mm), Yonge Reef, 14.61383°S, 145.6182°E, 18 February 2009, back reef, small coral rubble on sand, 15 m, stn LIZ09-10F, coll. MB-P & NLB (MTQ W34025).

*Also examined*. *Joeropsis salvati* Müller, 1989; holotype (microslides—SMF 17697) and paratypes (SMF 17690, part; ♀ 1.6 mm, 2 imm 1.2, 0.9 mm).

#### Description.

*Body* 3.6 as long as greatest width, dorsal surfaces matte, dull, moderately setose. *Cephalon* length 0.6 width, lateral margins converging anteriorly, smooth. *Pseudorostrum* 0.5 as long as proximal width, anterior margin rounded. *Eyes* lateral, with ~8 ommatidia, colour orange. *Pereonites* compact, close to each other, without dorsal carinae; *tergite lateral margin* subtruncate (those of pereonite 5 rounded), lateral margins smooth; median keels on sternites 5–7 (or on 6 and 7 or 7 only), keels weakly developed. *Pleotelson* width 1.1 length, dorsal surface with weak and indistinct sub-lateral ridges, caudomedial lobe narrowly rounded; lateral margins weakly convex, each with 5 spines.

*Antenna 1* with 5 articles; article 1 1.3 as long as wide, distolateral angle not lobed, weakly serrated, distomesial margin not serrate; article 2 0.5 as long as article 1, 1.2 as long as wide; lateral margins of articles 1 and 2 without cuticular scales; article 3 0.6 as long as article 2; article 4 0.8 as long as article 3; article 5 1.1 as long as article 3, 2.5 as long as proximal width, distally with 2 aesthetascs. *Antenna 2* peduncle article 5 4.2 as long as article 3, 1.9 as long as wide, lateral margin convex, with small cuticular scales, mesial margin straight; article 6 1.4 as long as width, distally expanded, distal width 2.3 proximal width, 0.6 as long as article 5, lateral margin without cuticular scales, mesial margin with 4 simple setae, distodorsal surface without setae; flagellum with 5 articles, article 1 1.1 as long as peduncle article 6, 2.6 as long as combined lengths of remaining articles.

*Mandible palp* article 2 with 2 long biserrate setae (terminally spatulate), article 3 with 5 long pectinate setae. Right incisor with symmetrical cusps, margins convex, distally acute; left incisor similar to right. *Molar process* distal half finely serrate. Right mandible spine row composed of 9 spines; left spine row divided by truncate lobe, without lacinoid spine. *Maxilla 1* lateral lobe with 12 strongly serrate RS; mesial lobe with 2 long, simple RS. *Maxilla 2* lateral lobe with 4 long, curved, finely serrate setae (2 short, 2 long); middle lobe with 4 long serrate setae, mesial lobe with 3 long simple setae and many long setules. *Maxilliped endite* 2 as long as greatest width, extending to middle of palp article 4, distal margin evenly rounded, mesially with 2 large serrations, with shallow distomesial concavity, with 4 mesial tubercular RS, distomesial margin with 3 coupling setae. *Maxilliped palp* article 2 2.3 as long as article 1, mesial lobe extending to distal margin of article 3, distomesial margin with 1 simple seta; article 3 0.5 as long as article 2, distomesial margin with 1 simple seta; article 4 4.6 as long as wide, mesial margin weakly concave, distally with 4 setae; article 5 0.2 as long as 4, with 4 terminal setae.

*Pereopod 1* basis 3.6 as long as wide, inferior margin with 1 proximal simple seta; ischium 0.7 as long as basis, 2.6 as long as wide; merus 0.7 length of ischium, 2.0 as long as wide; carpus 1.0 as long as ischium, 2.8 as long as wide; propodus 3.5 as long as wide, superior margin with 2 simple setae, inferior margin with 3 acute RS; dactylus 0.5 as long as propodus, with 2 claws. Pereopods 2–7 sub-similar, more slender than pereopod 1, each with 3 claws. *Pereopod 7* basis 2.7 as long as wide; superior margin with 1 short proximal simple seta; ischium 0.8 as long as basis, 3.1 as long as wide, superior margin strongly convex at midpoint, superior margin with 1 simple seta (distal margin with cuticular scale fringe), inferior distal angle with 1 seta; merus 0.6 as long as ischium, 1.9 as long as wide, superodistal angle with 2 simple setae (and short cuticular scale-spines); carpus 0.9 as long as ischium, 3.6 as long as wide, inferior margin with 7 setae, superior distal angle with 1 prominent pappose seta; propodus 1.1 as long as ischium, 3.7 as long as wide, inferior margin with 3 acute RS, superior margin with 6 simple setae; dactylus 0.4 as long as propodus.

*Pleopod 1* 2.6 as long as greatest width, lateral margin strongly concave, apical lobe broadly rounded, with long marginal setae, lateral margin with slender setae, distolateral lobe acute, not extending to distal margin. *Pleopod 2* protopod 2.3 as long as midwidth, lateral margin mid-half strongly convex, without setae, distal margin straight or weakly concave, with long marginal cuticular scales, apex narrowly rounded; *stylet* in retracted position extending beyond apex. *Pleopod 3* endopod 2.2 midwidth; exopod article 1 2.5 as long as wide, not extending to endopod apex, lateral margin fringed with cuticular scale-spines; article 2 0.5 as long as article 1, lateral and mesial margins with spine-like cuticular scale-setae (laterally; mesial with cuticular scale-setae).

*Uropod* peduncle extending slightly beyond margin of pleotelson, mediodistal corner strongly produced and acute, distolateral margin with 2 simple submarginal setae, mesial margin finely serrate. Exopod 0.7 as wide as endopod, 1.3 as long as wide, with 8 simple setae. Endopod 1.0 as long as wide, 0.3 as long as peduncle proximolateral margin, apex with 8 long simple setae.

**Female.** *Pleopod 2* 1.2 as long as proximal width, lateral margins strongly convex, posterior margins straight, apex with 3 sub-apical simple setae.

#### Size.

Males 1.1–1.6 mm, mean 1.4 mm (*n*=11); ovigerous females 1.4–1.8 mm, mean 1.5 mm (*n*=11), non-ovigerous females 1.1–1.7 mm, mean 1.4 mm (*n*=10); all from type series.

**Colour pattern.** All somites with dark brown chromatophores, darker and more dense on the anterior part of the head. In fresh specimens there is an anterior marginal band clear of chromatophores that together with the clear pseudorostrum, antenna 1 and antenna 2 gives the impression of a white margin to the front of the head. Some brown chromatophores are present on the female operculum.

#### Variation.

The number of pleotelson marginal teeth varied from 1 to 5 (*n*=24) in males with 4 (23%) or 5 (39%) most frequent; 4–6 (*n*=22) in ovigerous females with 5 (73%) most frequent and 4 and 6 occurring twice each.

#### Remarks.

Within the Great Barrier Reef and nearby regions *Joeropsis adusta* sp. n. can be identified by being entirely dark brown with a moderately setose dorsal surface, antennular article 1 without distolateral lobe, antenna 2 article 5 not expanded with weakly convex margins and an anteriorly rounded pseudorostrum.

There are a number of species of coral reef *Joeropsis*, some as yet undescribed, that are largely or entirely dark brown in colour. Among these are *Joeropsis salvati* Müller, 1989 from the Society Islands, *Joeropsis lentigo* Kensley & Schotte, 2002 from the Seychelles and *Joeropsis bicornis* Kensley, 2003 from the subtropical Easter Island.

*Joeropsis adusta* sp. n. differs from the closely similar *Joeropsis salvati* in the uropod having an apical spine (weak or absent in *Joeropsis salvati*, Fig. 4D), antenna 1 article 1 without distolateral lobe (with lobe), antenna 2 article 5 with straight mesial margin (angled), distally wide uropod (uropod margins sub-parallel) and the pseudorostrum anteriorly rounded (subtruncate to weakly concave, but variable). Müller (1989, fig. 78) illustrated the uropod as lacking a terminal spine, but that is not the case. Re-examinations of the holotype slides shows that the illustrated uropod is damaged, while the other uropod has a small terminal spine (Fig. 4D); a small spine is also present in two of the three paratypes examined (not visible in the third specimen).

**Figure 2.**
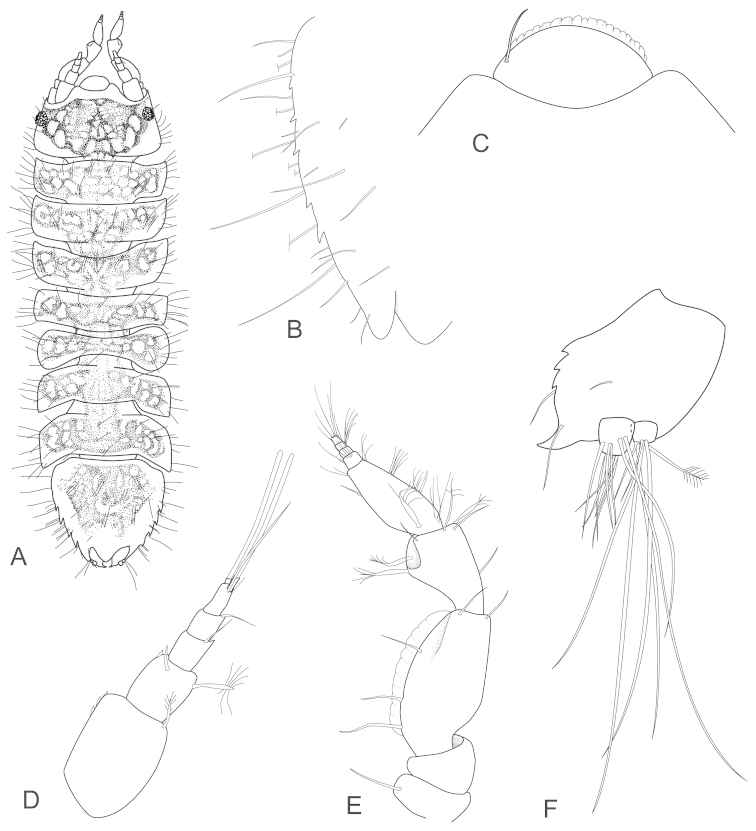
*Joeropsis adusta* sp. n. **A** holotype; remainder male paratype MTQ W33033. **A** dorsal view **B** pleotelson lateral margin **C** pseudorostrum **D** antenna 1 **E** antenna 2 **F** uropod.

**Figure 3.**
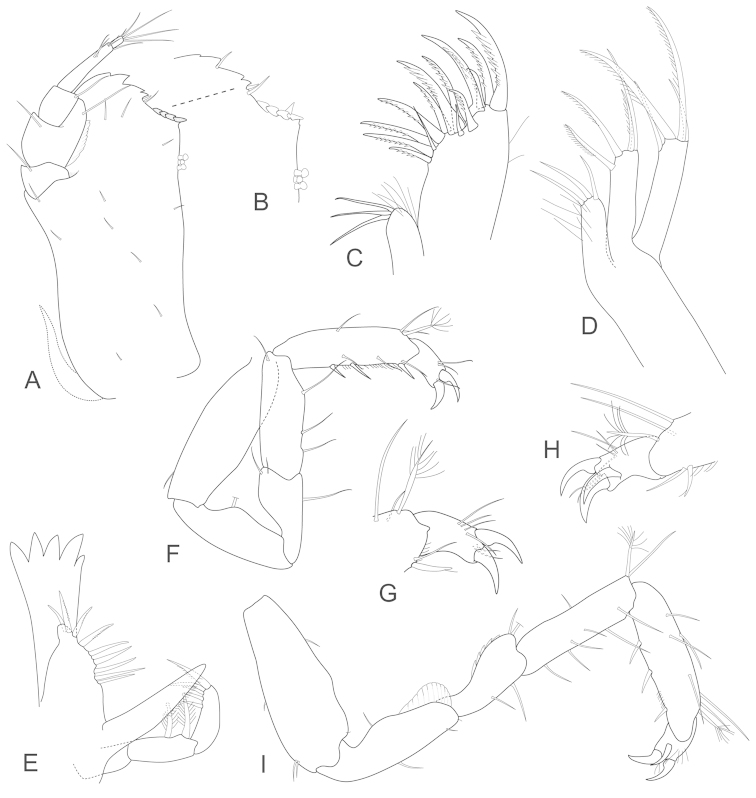
*Joeropsis adusta* sp. n. Male paratype MTQ W33033. **A** maxilliped **B** maxilliped, endite distomesial angle **C** maxilla **D** maxillula **E** mandible **F** pereopod 1 **G** pereopod 1, dactylus **H** pereopod 7 dactylus **I** pereopod 7.

**Figure 4.**
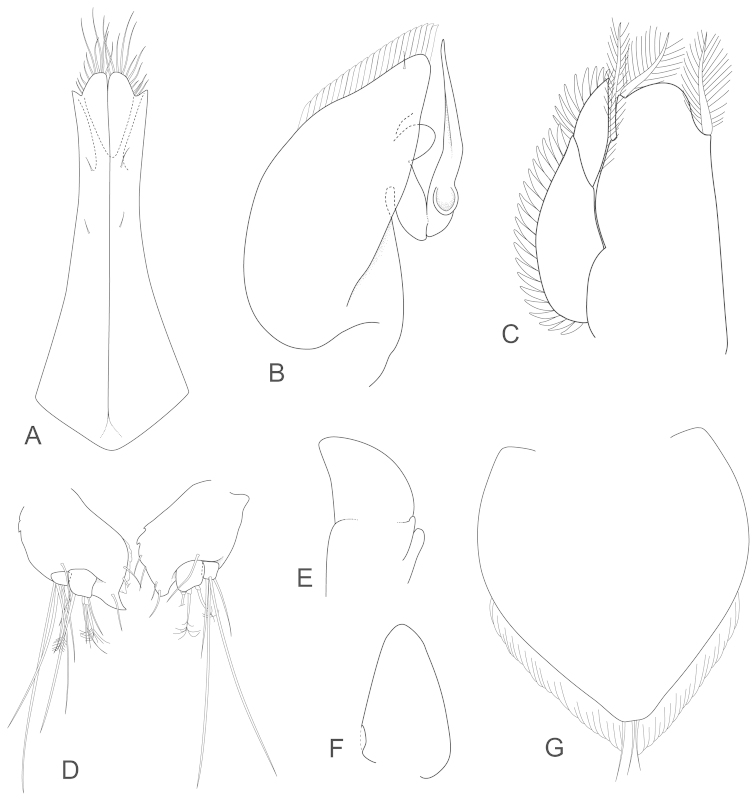
*Joeropsis adusta* sp. n. Male and female paratypes MTQ W33033. **A–C, E, F** pleopods 1–5 respectively **G** female pleopod 2 **D** *Joeropsis salvati* Müller, 1989, holotype, SMF 17690, uropods.

*Joeropsis bicornis* has a distinct head band, the brown chromatophores are more diffuse than in *Joeropsis adusta* and the uropod lacks a terminal spine (present in *Joeropsis adusta*).

*Joeropsis adusta* sp. n. is closely similar to *Joeropsis lentigo*, but morphological comparisons are not possible as the species was only briefly diagnosed with a figure of the habitus, and small figures of the male pleopod 1 and uropod, and there were no comparative comments. *Joeropsis adusta* differs in being dark brown (vs red–brown in *Joeropsis lentigo*), having a distally wider somewhat club-shaped uropod, the mesial margin of which is feebly serrate (smooth in *Joeropsis lentigo*), fewer marginal spines on the pleotelson (1–5 vs 3–6), more dense chromatophores (though this is probably variable) and a longer clear band on the anterior margin of the head.

*Joeropsis adusta* sp. n. is the only entirely dark-brown setose species recorded from the Lizard Island region, and fresh material can be identified on that basis. Other largely brown species are either more pale (e.g. *Joeropsis panstikta* sp. n., described herein), lack the abundant dorsal setae or have distinct clear areas such as a single pereonite (usually pereonite 5) or certain tergite lateral margin, or darker patches. Characters of the head, pleotelson spines, pseudorostrum shape, setosity and details of the appendages should all be compared to other species where possible.

#### Distribution.

Fringing reef at North Point and Seabird Islet, Lizard Island, South Direction Island and back reef of Yonge Reef (Fig. 1); 1 to 25 metres.

#### Etymology.

The epithet taken from the Latin *adustus*, meaning singed or scorched (to brown).

### Joeropsis goobita sp. n.

Taxon classificationAnimaliaIsopodaJoeropsididae

http://zoobank.org/3B2755A8-6C20-4A50-918F-191B65240AD3

Figs 5 , 6 , 7

#### Material.

*Holotype*. ♂ (1.6 mm), Seabird Islet, Lizard Island, 14.69497°S, 145.4657°E, 23 February 2009, outer reef front, dead coral heads, 6–8 m, stn LIZ09-19B, coll. MB-P & NLB (MTQ W31841).

*Paratypes*. 4 ♂ (1.6, 1.6, 1.5 [dissected, 3 slides], 1.5, 1.3 mm), 8 ♀ (ovig. 1.6 [dissected, 2 slides], non-ovig. 1.7, 1.4, 1.5, 1.5, 1.3, 1.0. 0.9 mm), same data as holotype (MTQ W31251). ♂ (1.6 mm), ♀ (non-ovig. 1.3 mm), North Point, Lizard Island, 14.64553°S, 145.45335°E, 12 April 2008, compacted dead *Acropora*, 0.5 m, stn CGLI-20C, coll. NLB (MTQ W13977). ♂ (1.6 mm), Seabird Islet, in from lagoon entrance, 14.68900°S, 145.46710°E, patch reef, 11 April 2008, dead coral heads 1.0–2.0 m, stn CGLI-18A, coll. NLB & MB-P. (MTQ W31842). ♂ (1.7 [whole mount], 1.6, 1.5, 1.5 mm), ♀ (non-ovig. 1.6, 1.6, 1.5 mm), juv. (1.0 mm), Yonge Reef, 14.62317°S, 145.6201°E, 13 February 2009, reef pass, dead coral heads, 5 m, stn LIZ09-11A, coll. NLB & MB-P (MTQ W31843). ♂ (1.4, 1.3 mm), ♀ (ovig. 1.6, 1.4, 1.3, non-ovig. 1.6, 1.5 mm), Day Reef, 14.48356°S, 145.5459°E, 13 February 2009, outer reef, coral heads in gully, 10 m, LIZ09-04B, coll. MB-P. (MTQ W31844).

*Additional material*. 5, not measured, Day Reef, 14.47119 °S, 145.5297°E, 13 February 2009, outer reef, dead coral on vertical wall, 10–12 m, stn LIZ09-03A, coll. MB-P (MTQ W31845). ♂ (in 2 pieces), Day Reef, 14.48283°S, 145.5564°E, 19 February 2009, outer reef front, dead *Acropora* slab, 10 m, stn LIZ09-13C, coll. NLB & MB-P (MTQ W31846). 1, Yonge Reef, 14.57735°S, 145.61050°E, 10 September 2010, lee side (western) of reef, coral rubble, 10 m, stn LI10-127A, coll. CB (MTQ W33716)

#### Description.

*Body* 2.6 as long as greatest width, appearing dorso-ventrally flat, dorsal surfaces matte, dull, moderately setose. *Cephalon* length 0.5 width, lateral margins converging anteriorly or weakly concave, finely serrate (posterior half). *Pseudorostrum* 0.5 as long as proximal width, anterior margin rounded. *Eyes* lateral, with 12 ommatidia, colour orange (when live). *Pereonites* not compact, widely spaced, without dorsal carinae; *tergite lateral margin* subtruncate, lateral margins smooth; median keels weak, not carinate. *Pleotelson* width 1.4 length; dorsal surface with single median and paired submedian low ridges, caudomedial lobe sub-acute; lateral margins convex, each with 5–6 spines.

*Antenna 1* with 5 articles; article 1 1.3 as long as wide, distolateral angle not lobed, not serrated, distomesial margin not serrate; article 2 0.5 as long as article 1, 1.2 as long as wide; lateral margins of articles 1 and 2 without cuticular scales; article 3 0.4 as long as article 2; article 4 1.1 as long as article 3; article 5 2.4 as long as article 3, 3 as long as proximal width, distally with 2 aesthetascs. *Antenna 2* article 5 3.8 as long as article 3, 1.6 as long as wide, lateral margin strongly convex, with prominent cuticular scales, mesial margin straight; article 6 1.5 as long as width, distally expanded, distal width 2.2 proximal width, 0.6 as long as article 5, lateral margin with cuticular scales on distal one-third, mesial margin with 3 simple setae, distodorsal surface without setae; antenna 2 flagellum with 6 articles, article 1 1.1 as long as peduncle article 6, 2.2 as long as combined lengths of remaining articles.

*Mandible palp* article 2 with 2 long biserrate setae, article 3 with 3 long pectinate setae. Right incisor with symmetrical cusps, margins convex, distally acute; left mandible incisor similar to right incisor. *Molar process* distal half finely serrate. Right mandible spine row composed of 7 spines; left spine row without lacinoid spine. *Maxilla 1* (not figured) lateral lobe with 9 strongly serrate RS, and 3 simple RS; mesial lobe with 5 long, simple RS. *Maxilla 2* (not figured) lateral lobe with 4 long, curved, finely serrate setae (2 short, 2 long); middle lobe with 4 long serrate setae (2 short, 2 long), mesial lobe with 4 long simple setae and many long setules. *Maxilliped endite* 2 as long as greatest width, extending beyond palp, distal margin evenly rounded, smooth, with shallow distomesial concavity, with 4 mesial tubercular RS, distomesial margin with 3 coupling setae. *Maxilliped palp* article 2 1.6 as long as article 1, mesial lobe absent, distomesial margin with 1 simple setae; article 3 0.6 as long as article 2, distomesial margin with 4 simple setae; article 4 3.1 as long as wide, mesial margin weakly concave, distally with 4 setae; article 5 0.2 as long as 4, with 4 terminal setae. *Epipod* 2.3 as long as basal width, distally narrowly rounded; 0.8 as long as palp, 0.4 as long as endite.

*Pereopod 1* basis 2.3 as long as wide, inferior margin with 1 simple seta; ischium 0.8 as long as basis, 2.6 as long as wide; merus 0.7 length of ischium, 1.5 as long as wide; carpus 1.0 as long as ischium (1.04), 2.1 as long as wide; propodus 3.1 as long as wide, superior margin with 2 simple setae (and prominent penicillate seta at distal angle); inferior margin with 2 acute RS, dactylus 0.5 as long as propodus, with 2 claws. Pereopods 2–7 sub-similar, more slender than pereopod 1, each with 3 claws. *Pereopod 7* basis 2.9 as long as wide; superior margin with 2 short simple setae; ischium 0.8 as long as basis, 2.7 as long as wide, superior margin weakly convex at midpoint, superior margin with 2 simple setae, inferior distal angle with 0 setae; merus 0.7 as long as ischium, 1.8 as long as wide, superodistal angle with 2 simple setae; carpus 0.9 as long as ischium, 3.2 as long as wide, inferior margin with 2 setae (distal one-third with cuticular scale-setae), superior distal angle with 1 prominent pappose seta; propodus 1.2 as long as ischium, 4.5 as long as wide, inferior margin with 2 acute RS, superior margin with 2 simple setae (and distal penicillate seta); dactylus 0.3 as long as propodus.

*Pleopod 1* 2.2 as long as greatest width, lateral margin strongly concave, apical lobe broadly rounded, with long marginal setae, lateral margin with slender setae, distolateral lobe narrowly rounded, not extending to distal margin. *Pleopod 2* protopod 2 as long as midwidth, lateral margin mid-half weakly convex, without setae, distal margin weakly concave, with long marginal cuticular scales, apex acute; *stylet* in retracted position not reaching apex. *Pleopod 3* endopod 1.2 midwidth (medially fused to protopod); exopod article 1 3.0 as long as wide, not extending to endopod apex, lateral margin weakly fringed with cuticular scale-setae; article 2 0.4 as long as article 1, lateral margin with short cuticular scale-setae.

*Uropod* peduncle extending well beyond margin of pleotelson, mediodistal corner weakly produced and acute, distolateral margin 1 simple submarginal setae, mesial margin smooth. Exopod 0.5 as wide as endopod, 1.5 as long as wide, with 5 simple setae. Endopod 1.3 as long as wide, 1.0 as long as peduncle proximolateral margin, apex with 5 long simple setae.

**Female.** *Pleopod 2* 1.3 as long as proximal width, lateral margins weakly convex, posterior margins weakly sinuate, with long cuticular scale-setae, apex with 2 sub-apical simple setae.

#### Colour pattern.

Head and pereonites 1–4, 6, 7 and pleotelson with loosely spread chromatophores, pereonite 5 clear; pereonites 4 and 6 slightly darker than other pereonites in some specimens. If chromatophores are contracted specimens appear more pale than figured specimen.

#### Size.

Males 1.3–1.7 mm (mean= 1.5 mm, *n*=14); ovigerous females 1.3–1.6 mm (mean= 1.5 mm, *n*=4), non-ovigerous females 0.9–1.6 mm (mean= 1.4 mm, *n*=12).

#### Remarks.

*Joeropsis goobita* sp. n. may be recognized by the setose body surfaces, the relatively short (0.5 long as wide) and anteriorly narrowed head, loosely scattered chromatophores over the dorsum giving a finely spotted and translucent appearance, except for pereonite 5, which is clear; there is no distinct head band and the pseudorostrum is short and anteriorly rounded. The body is noticeably flat in appearance.

**Figure 5.**
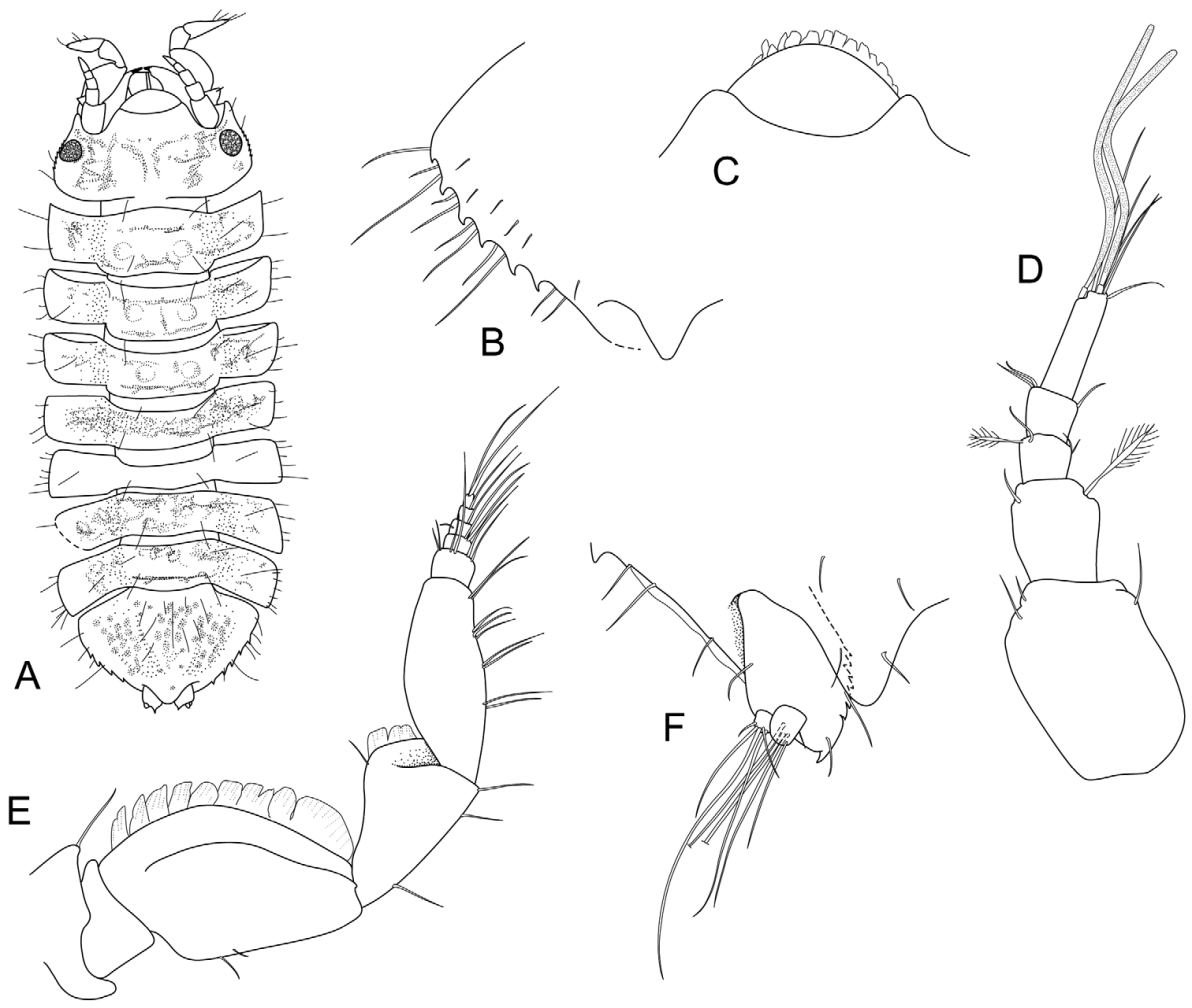
*Joeropsis goobita* sp. n. **A** holotype; remainder male paratype (1.7 mm MTQ W31843). **A** dorsal view **B** pleotelson lateral margin **C** pseudorostrum **D** antenna 1 **E** antenna 2 **F** uropod.

**Figure 6.**
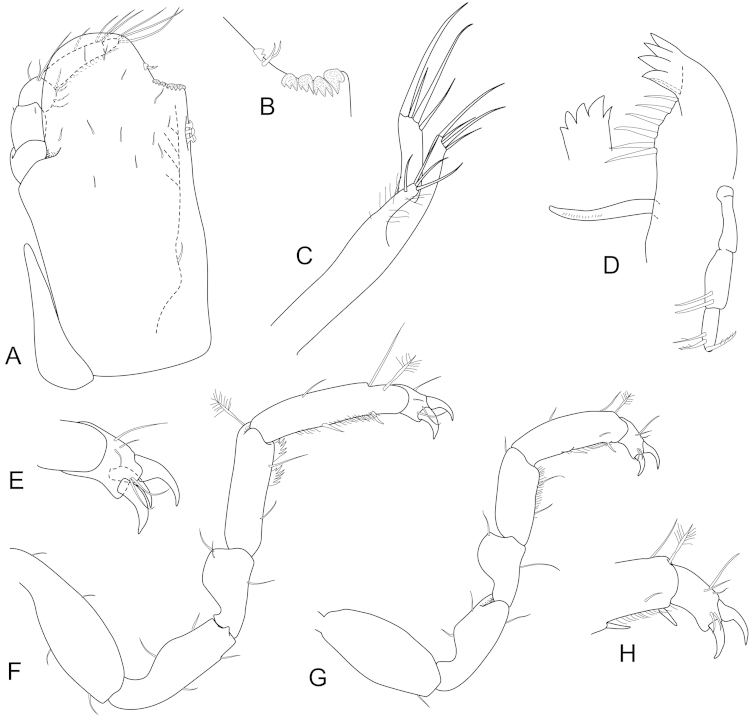
*Joeropsis goobita* sp. n. **A–D** female paratype (ovig. 1.6 mm) **E–H** male paratype (1.6 mm MTQ W31251). **A** maxilliped **B** maxilliped, endite distomesial angle **C** maxilla **D** mandible **E** pereopod 7 dactylus **F** pereopod 7 **G** pereopod 1, **H** pereopod 1 dactylus.

**Figure 7.**
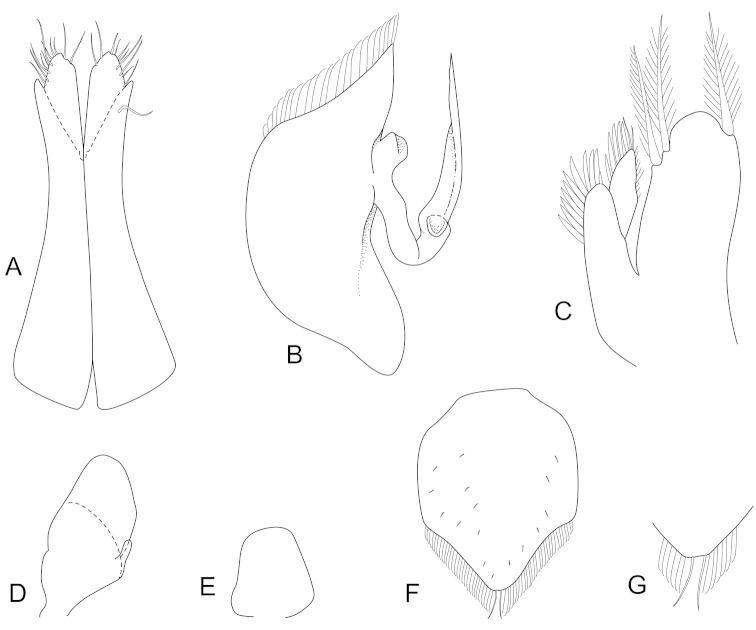
*Joeropsis goobita* sp. n. **A–C** male paratype (1.5 mm) **D–E** female paratype (1.7 mm MTQ W31251). **A–E** pleopods 1–5 respectively **F** female pleopod 2 **G** female pleopod 2 apex.

#### Distribution.

North Point, Lizard Island and off Seabird Islet; Day and Yonge Reefs (Fig. 1); depths probably intertidal to 12 m.

#### Etymology.

The epithet is an Aboriginal word meaning small; noun in apposition.

### Joeropsis jiigurru sp. n.

Taxon classificationAnimaliaIsopodaJoeropsididae

http://zoobank.org/38472B0C-31F5-4A8A-A615-7B84A9FEB407

Figs 8 , 9 , 10

#### Material.

All material from the Lizard Island region, northern Great Barrier Reef, Queensland.

*Holotype*. ♂ (1.9 mm), Hicks Reef, 14.44803°S, 145.4992°E, 21 February 2009, outer reef front, coral rubble under bommie overhang, 15 m, stn LIZ09-16B, coll. NLB & MB-P (MTQ W33666).

*Paratypes*. ♀ (ovig. 1.3 mm), Day Reef, 14.48539°S, 145.5464°E, 19 February 2009, outer reef front, coral rubble in gully, 17 m, stn LIZ09-12F, coll. NLB & MB-P (MTQ W33667). ♂ (1.9 mm), Yonge Reef, 14.57735°S, 145.61050°E, 10 September 2010, inner reef front, silty coral rubble under bommie overhang, 5 m, stn LI10-127B, coll. CB (MTQ W33668).

#### Description.

*Body* 2.9 as long as greatest width, dorsal surfaces polished in appearance, without setae. *Cephalon* length 0.7 width, lateral margins straight, finely serrate (proximal half). *Pseudorostrum* 1.2 as long as proximal width, anterior margin acute (lateral margin anteriorly concave). *Eyes* sublateral, with 4 ommatidia, colour dark brown. *Pereonites* compact, close to each other (posteriorly compact, anteriorly spaced), without dorsal carinae; *tergite lateral margin* subtruncate (tergite lateral margin 1–5 serrate), lateral margins 1–4 finely serrate; median keels on sternites 1–6, keels well developed (on sternites 1–3; serrate). *Pleotelson* width 0.9 length; dorsal surface with single median and paired submedian low ridges, caudomedial lobe broadly rounded; lateral margins weakly convex, each with 8 spines.

*Antenna 1* with 5 articles; article 1 1.2 as long as wide, distolateral angle strongly lobed, strongly serrated, distomesial margin not serrate; article 2 0.8 as long as article 1, 0.8 as long as wide; lateral margins of articles 1 and 2 with cuticular scales on distal margins; article 3 0.4 as long as article 2; article 4 0.4 as long as article 3; article 5 1.3 as long as article 3, 1.6 as long as proximal width, distally with 3 aesthetascs. *Antenna 2* peduncle article 5 1.7 as long as articles 1–4 combined, 2.9 as long as article 3, 1.7 as long as wide, lateral margin convex, with prominent cuticular scales, mesial margin weakly convex; article 6 1.7 as long as width, distally expanded, distal width 2.2 proximal width, 0.7 as long as article 5, lateral margin without cuticular scales, mesial margin with 10 simple setae, distodorsal surface without setae; flagellum with 6 articles, article 1 0.7 as long as peduncle article 6, 1.6 as long as combined lengths of remaining articles.

*Mandible palp* article 2 with 3 long biserrate setae, article 3 with 5 long pectinate setae. Right incisor with 5 cusps (4 large, 1 small), margins convex, distally acute; left mandible incisor similar to right incisor. *Molar process* distal quarter finely serrate. Right mandible spine row composed of 8 spines; left spine row not divided by truncate lobe, without lacinoid spine. *Maxilliped* endite 2.3 as long as greatest width, extending to distal margin of palp article 4, distal margin evenly rounded, smooth, with shallow distomesial concavity, with 3 mesial tubercular RS (and 1 triangular RS), distomesial margin with 3 coupling setae. *Maxilliped palp* article 2 4.8 as long as article 1, mesial lobe extending to distal margin of article 3, distomesial margin with 1 simple seta; article 3 0.4 as long as article 2, distomesial margin with 2 simple setae; article 4 2.9 as long as wide, mesial margin weakly concave, distally with 2 setae; article 5 0.1 as long as 4, with 7 terminal setae. *Epipod* 4.3 as long as basal width, distally narrowly rounded; 1.0 as long as palp, 0.4 as long as endite.

*Pereopod 1* basis 4.4 as long as wide, inferior margin with 2 simple setae; ischium 0.6 as long as basis, 2.3 as long as wide; merus 0.6 length of ischium, 0.5 as long as wide; carpus 1.0 as long as ischium, 5.1 as long as wide; propodus 5.6 as long as wide, superior margin 2 simple setae; inferior margin with 2 acute RS, dactylus 0.4 as long as propodus, with 2 claws. Pereopods 2–7 sub-similar, more slender than pereopod 1, each with 2 claws. *Pereopod 7* basis 3.2 as long as wide; superior margin with 1 short simple seta; ischium 0.8 as long as basis, 3.1 as long as wide, superior margin weakly convex at midpoint, superior margin with 2 simple setae (and cuticular scales), inferior distal angle with 0 setae; merus 0.6 as long as ischium, 2.3 as long as wide, superodistal angle with 2 simple setae; carpus 1.0 as long as ischium, 5.5 as long as wide, inferior margin with 2 setae, superior distal angle with 1 prominent pappose seta; propodus 1.0 as long as ischium, 6.3 as long as wide, inferior margin with 2 acute RS, superior margin with 1 simple setae (and distal penicillate seta); dactylus 0.4 as long as propodus.

*Pleopod 1* 2.4 as long as greatest width, lateral margin weakly concave, apical lobe broadly rounded, with short marginal setae, lateral margin with slender setae, distolateral lobe narrowly rounded, not extending to distal margin. *Pleopod 2* protopod 2.6 as long as midwidth, lateral margin mid-half weakly convex, without setae, distal margin weakly convex, without marginal cuticular scales, apex broadly rounded; *stylet* in retracted position extending beyond apex. *Pleopod 3* endopod 2.4 midwidth; exopod article 1 3.6 as long as wide, extending to endopod apex, lateral margin densely fringed with cuticular scale-setae; article 2 0.5 as long as article 1, lateral and mesial margins with short cuticular scale-setae and with spine-like cuticular scale-setae (spine-like distally only).

*Uropod* peduncle extending slightly beyond margin of pleotelson, mediodistal corner weakly produced and acute, distolateral margin with 4 simple submarginal setae, mesial margin finely serrate. Exopod 0.7 as wide as endopod, 1.1 as long as wide, with 5 simple setae. Endopod 0.8 as long as wide, 0.3 as long as peduncle proximolateral margin, apex with 9 long simple setae.

**Female.** *Pleopod 2* 1.3 as long as proximal width with weak median longitudinal carina, lateral margins straight, posterior margins straight, with cuticular scales.

#### Colour pattern.

White with narrow transverse red–brown band on head and posterior of pereonites 1, 2 and 4; interocular head band occupies 10% of head length.

#### Size.

Males 1.9 mm, ovigerous female 1.3 mm.

#### Remarks.

*Joeropsis jiigurru* sp. n. is most readily recognized by the distinctive colour pattern of four narrow transverse bands, running between the eyes and across pereonites 1, 2 and 4. The eyes are noticeably small, the lateral margins of the head and tergite lateral margin 2–4 are serrate, the pleotelson is relatively broad, with eight well-developed teeth on each lateral margin, the pseudorostrum is anteriorly acute, and the pereopods are relatively slender, with all pereopods with two dactylar claws. The male holotype has well-developed serrated mid-sternal keels—an apparently unique character for the genus.

**Figure 8.**
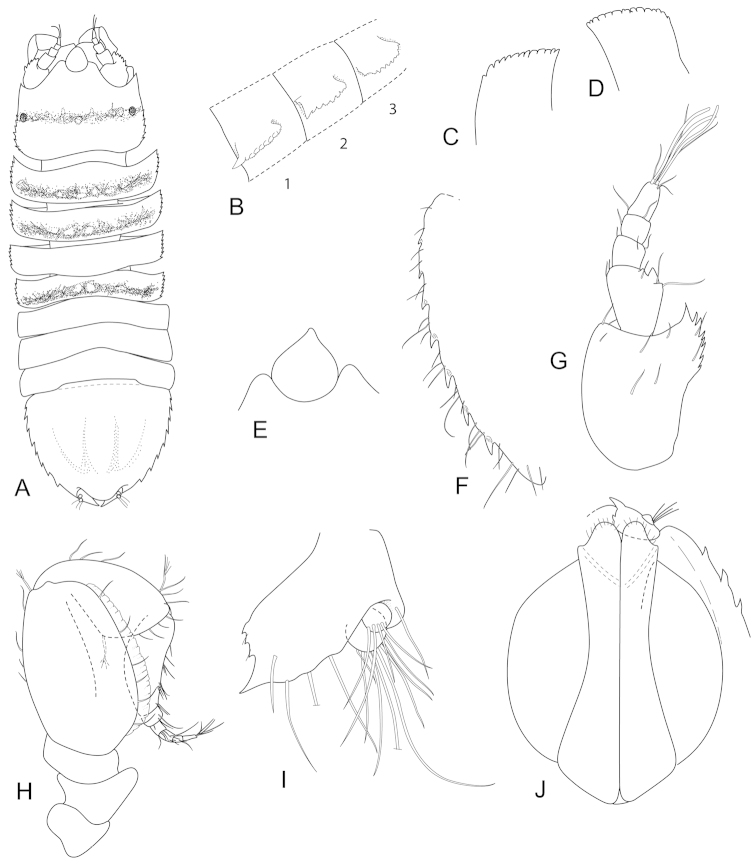
*Joeropsis jiigurru* sp. n. **A–F, J** holotype; remainder male paratype (1.9 mm) MTQ W33668. **A** dorsal view **B** median keels on sternites 1–3 on pleotelson lateral margin **C** coxa 2, **D** coxa 6 **E** pseudorostrum **F** pleotelson lateral margin **G** antenna 1 **H** antenna 2 **I** uropod **J** pleopods 1 and 2, *in situ*.

**Figure 9.**
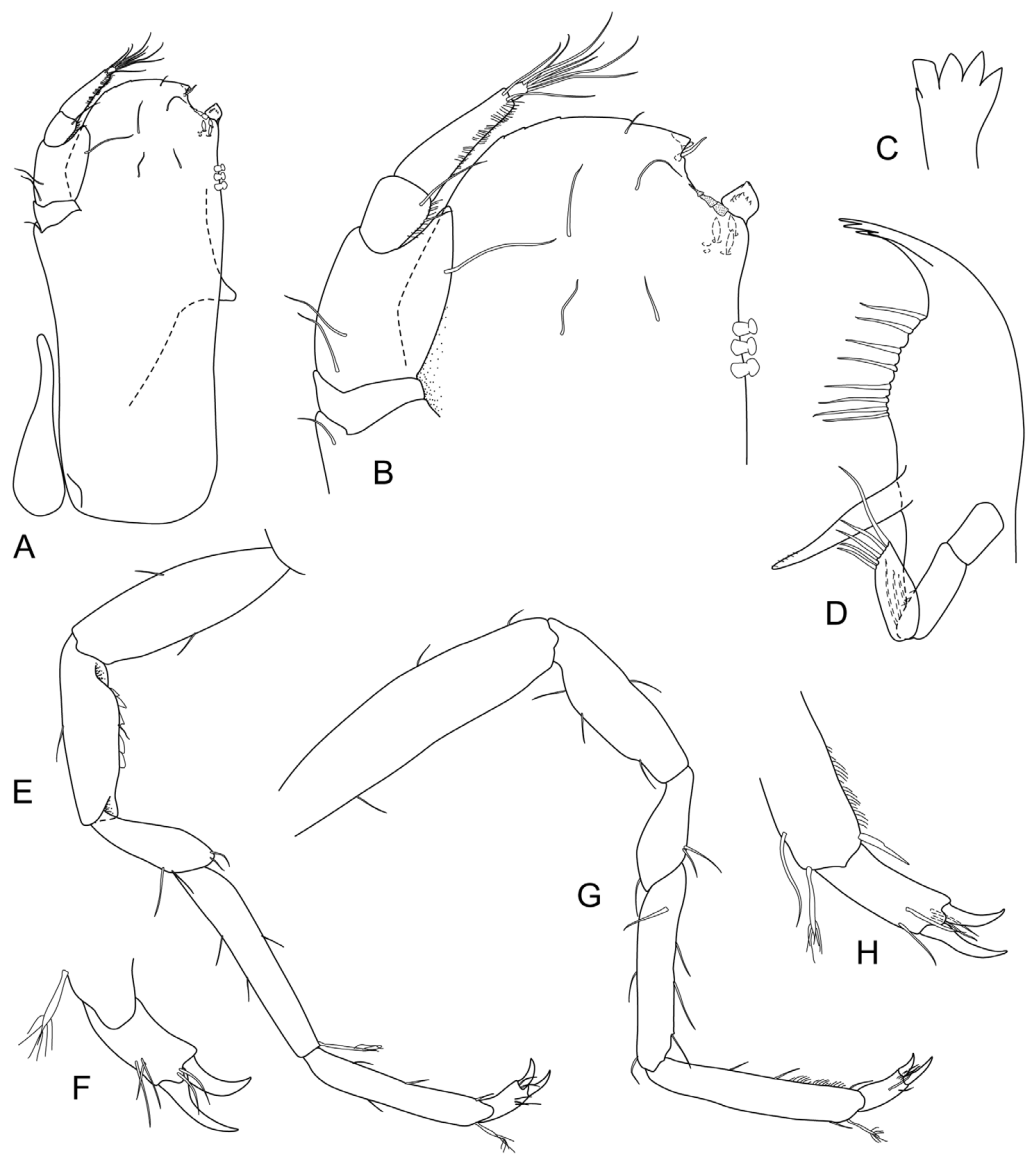
*Joeropsis jiigurru* sp. n. Female paratype MTQ W33667. **A** maxilliped **B** maxilliped, endite distomesial angle **C** left mandible incisor **D** right mandible maxilla maxillula **E** pereopod 7 **F** pereopod 7 dactylus **G** pereopod 1, **H** pereopod 1 dactylus.

**Figure 10.**
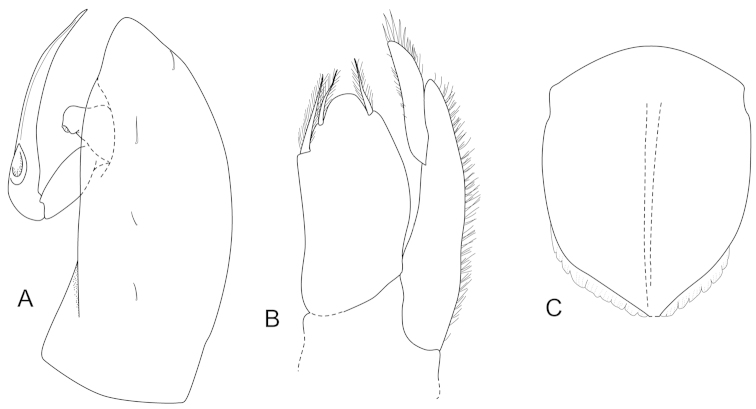
*Joeropsis jiigurru* sp. n. Male paratype MTQ W33668. **A** pleopod 2 **B** pleopod 3 **C** female paratype MTQ W33667, pleopod 2.

#### Distribution.

Lizard Island region: Hicks, Day and Yonge Reefs (Fig. 1); at depths 5 to 17 metres.

#### Etymology.

The epithet is the Aboriginal name for Lizard Island in the language of the Dingaal people (DERM Lizard Island http://www.derm.qld.gov.au/parks/lizard-island/culture.html); noun in apposition.

### Joeropsis makrogenys sp. n.

Taxon classificationAnimaliaIsopodaJoeropsididae

http://zoobank.org/F1B1094E-2FFA-47BB-B925-401D8197AC7A

Figs 11 , 12 , 13

Joeropsis sandybrucei . – Bruce 2009b: 805 (part, Lizard Island specimens).

#### Material.

*Holotype*. ♂ (3.0 mm), ♀ (ovig. 2.5 mm), Lizard Island, 14.6867°S, 145.4551°E, 30 August 2010, 3 m, shallow lagoon, LI10-037, coll. I. Marin (MTQ W32671).

*Paratypes*. 3 ♂ (2.3 mm [dissected]; imm. 1.2 1.1 mm), 4 ♀ (non-ovig. 2.5 [dissected], 2.2, 2.0, 1.9 mm), 5 mancas (1.2, 1.2 1.1, 1.1, 1.0 mm), ‘High Rock’, east of South Direction Island, 14.82428°S, 145.55270°E, 11 September 2010, reef slope, clean coral rubble, 6 m, LI10-134C, coll. CB (MTQ W34010). ♂ (2.2 mm), ♀ (ovig. 3.1, non-ovig. 2.5 mm), Hicks Reef, 14.44803°S, 145.4992°E, 21 February 2009, outer reef front, dead coral heads on reef edge, 5–7 m, LIZ09-16E, coll. NLB & MB-P (MTQ W31289). ♂ (1.3 mm), Yonge Reef, 14.62317°S, 145.6201°E, 13 February 2009, reef pass, dead coral heads, 5 m, stn LIZ09-11A, coll. NLB & MB-P (MTQ W34011). ♀ (non-ovig. 1.3 mm), Day Reef, 14.48283°S, 145.5564°E, 19 February 2009, outer reef front, small rubble in gully, 7.5 m, stn LIZ09-13A, coll. MB-P & NLB (MTQ W34012). ♀ (non-ovig. 2.3 mm), Yonge Reef, 14.60681°S, 145.6311°E, 20 February 2009, outer reef front, dead coral, 30 m, stn LIZ09-15B, coll. S. Smith & JC (MTQ W31286). ♂ (2.1, imm 1.1 mm), Hicks Reef, 14.44803°S, 145.4992°E, 21 February 2009, outer reef front, dead coral heads on reef edge, 5–7 m, LIZ09-16E, coll. NLB & MBP (MTQ W34013). ♀ (ovig. 2.3 mm), MacGillivray Reef, 14.64792°S, 145.48660°E, 1 September 2010, 14 m, reef slope, coral rubble, LI10-041, coll. M. Capa (MTQ W32691). ♂ (1.6 mm), Day Reef, 14.47045°S, 145.52840°E, 5 September 2010, 17 m, outer reef, dead coral heads, LI10-077B, coll. CB (MTQ W34014). ♀ (non-ovig. 2.6 mm), Lizard Island, 14.6867°S, 145.4551°E, 30 August 2010, 3 m, shallow lagoon, LI10-037, coll. I. Marin (MTQ W34015). ♀ (ovig. 2.8 mm), north side of Lizard Island, ‘Washing Machine’, 14.65383°S, 145.46340°E, 27 August 2010, coral rubble, 5 m, LI10-017C, coll. CB (MTQ W34016). ♀ (non-ovig 3.0 mm), imm/manca (0.9 mm), Lizard Island, ‘Bommie Bay’, 14.66157°S, 145.46160°E, 8 August 2010, 12 m, coral rubble, LI10-100A, coll. CB (MTQ W34017). ♂ (2.2 mm), imm. (1.3 mm), Lizard Island, ‘Bommie Bay’, 14.66157°S, 145.47160°E, 8 September 2010, 8 m, dead coral heads, LI10-101B, coll. CB (MTQ W34018). ♀ (non-ovig. 2.1 mm), Lizard Island, ‘Bommie Bay’, 14.6615°S, 145.4717°E, 9 September 2010, 10 m, silty coral in cave, LI10-111, coll. M. Wakeford (MTQ W32952).

*Additional material*. Lizard Island, non-type specimens from Bruce (2009): 2 ♂ (2.3, 2.1 mm), 8 ♀ (ovig. 3.9, 3.6, 3.5, 3.2, 3.2, 2.8; non-ovig. 3.2, 3.1 mm), imm. (1.2 mm), 14.6890°S, 145.4671°E, patch reef, lagoon entrance in from Seabird Islet, 11 April 2008, dead coral heads, 1.0–2.0 m, stn CGLI-018A, coll. NLB & MB-P (MTQ W13805). ♀ (imm. 1.6 mm), patch reef, in lagoon entrance from Seabird Islet, 14.6890°S, 145.4671°E, 19 April 2008, medium small rubble, 1.0–3.0 m, CGLI-041B, coll. NLB (MTQ W13806). ♂ (1.6 mm), ♀ (ovig. 2.6 mm), North Point, 14.64553°S, 145.45335°E, 12 April 2008, dead coral heads, 1.0–1.5 m, CGLI-20A, coll. NLB (MTQW13807). ♂ (2.0 mm), ♀ (ovig. 2.9 mm), patch reef off LIRS, Casuarina Beach, 14.68039°S, 145.44530°E, 15 April 2008, dead corals, 1.0 m, CGLI-031B, coll. NLB (MTQ W13808). **Papua New Guinea, Madang:** 19 ♂, ♀ and imm., 5°08.4’S, 145°50.9’E, 28 April 1989, south of Wongat Island, barrier reef back reef, coarse *Halimeda* covered rubble, 7 m, stn. 14B, coll. NLB & Rosella Ueba (MTQ W19759). 1 specimen, Wongat Island, 5°08.1’S, 145°50.6’E, 1 May 1989, semi-exposed, rubble among alcyonarians, NW corner, 1–2 m, Stn 16, coll. NLB (MTQ W19769). 1 specimen, Masamoz Reef, 5°08.1’S, 145°50.3’E, 24 April 1989, dead plates and *Acropora* rubble, 12 m, stn 10A, coll. NLB & M. Jebb (MTQ W19772).

*Also examined*. *Joeropsis indica* Müller, 1991b, holotype (specimen lacking head and pleotelson + microslide) (SMF 18191) and female paratype (microslide only) (SMF 18192), Beruwala [approx. 37 km south of Colombo], Sri Lanka, 8–16 May 1989, coll. H.-G. Müller.

#### Description.

*Body* 3.4 as long as greatest width, dorsal surfaces finely granular, with few setae. *Cephalon* length 0.7 width, lateral margins weakly sinuate, smooth. *Pseudorostrum* 0.4 as long as proximal width, anterior margin concave. *Eyes* lateral, with 12–16 ommatidia, colour black. *Pereonites* not compact, widely spaced, without dorsal carinae; *tergite lateral margin* subtruncate, lateral margins smooth; median keels weak, not carinate. *Pleotelson* width 1.2 length, dorsal surface with single median and paired submedian low ridges, caudomedial lobe narrowly rounded; lateral margins convex, each with 6 spines.

*Antenna 1* with 6 articles; article 1 1.4 as long as wide, distolateral angle strongly lobed, strongly serrated, distomesial margin not serrate; article 2 0.5 as long as article 1, 1.3 as long as wide; lateral margins of articles 1 and 2 without cuticular scales; article 3 0.7 as long as article 2; article 4 0.6 as long as article 3; article 5 0.4 as long as article 3, 1.0 as long as proximal width, distally with 2 aesthetascs. *Antenna 2* peduncle article 5 1.9 as long as articles 1–4 combined, 5.3 as long as article 3, 2.1 as long as wide, lateral margin distal three-quarters straight, with prominent cuticular scales, mesial margin angled; article 6 1.3 as long as width, distally expanded, distal width 2.1 proximal width, 0.5 as long as article 5, lateral margin without cuticular scales, mesial margin with 3 simple setae, distodorsal surface with few simple setae; flagellum with 6 articles, article 1 1.1 as long as peduncle article 6, 4 as long as combined lengths of remaining articles.

*Mandible palp* article 2 with 3 long biserrate setae, article 3 with 5 long pectinate setae. Left mandible incisor with lateral cusp thick, produced, distally truncate and mesial cusps rounded, ‘fish-tail’ shape; right incisor with symmetrical cusps, margins convex, distally acute and mesial-most cusp wide, distally subtruncate. *Molar process* distal half finely serrate. Right mandible spine row composed of 6 spines; left mandible spine row composed of 9 spines, not divided by truncate lobe; with lacinoid spine. *Maxilla 1* lateral lobe with 10 strongly serrate RS, and 3 simple RS; mesial lobe with 3 long, simple RS. *Maxilla 2* lateral lobe with 4 long, curved, finely serrate setae (2 short, 2 long); middle lobe with 4 long serrate setae (2 short, 2 long), mesial lobe with 4 long simple setae and many long setules. *Maxilliped endite* 2.1 as long as greatest width, extending to distal margin of palp article 3, distal margin entirely oblique, smooth, without distomesial concavity, with 4 mesial tubercular RS, distomesial margin with 3 coupling setae. *Maxilliped palp* article 2 2.6 as long as article 1, mesial lobe short, distomesial margin with 2 simple setae; article 3 0.7 as long as article 2, distomesial margin with 3 simple setae; article 4 2.7 as long as wide, mesial margin weakly concave, distally with 5 setae; article 5 0.2 as long as 4, with 8 terminal setae.

*Pereopod 1* basis 2.3 as long as wide, inferior margin with 4 simple setae (short) inferior margin with 1 simple seta; ischium 0.7 as long as basis, 2.2 as long as wide; merus 0.7 length of ischium, 1.4 as long as wide; carpus 1.1 as long as ischium, 2.7 as long as wide; propodus 4.4 as long as wide, superior margin 4 simple setae; inferior margin with 3 acute RS, dactylus 0.5 as long as propodus, with 2 claws (and acute robust seta). Pereopods 2–7 sub-similar, more slender than pereopod 1, each with 3 claws. *Pereopod 7* basis 3.2 as long as wide; superior margin with 7 short simple setae, inferior margin with 2 simple setae; ischium 0.6 as long as basis, 2.5 as long as wide, superior margin convex, with 1 simple seta, inferior distal angle with 1 seta; merus 0.6 as long as ischium, 1.6 as long as wide, superodistal angle with 1 simple seta (superior margin with weak cuticular scales); carpus 1.1 as long as ischium, 3.3 as long as wide, inferior margin with 4 setae, superior distal angle with 2 prominent setae; propodus 1.1 as long as ischium, 3.7 as long as wide, inferior margin with 3 acute RS, superior margin with 4 simple setae (3 short, 1 long); dactylus 0.4 as long as propodus.

*Pleopod 1* 2.3 as long as greatest width, lateral margin strongly convex, apical lobe broadly rounded, with short and long marginal setae, lateral margin with slender setae, distolateral lobe acute, not extending to distal margin. *Pleopod 2* protopod 2.3 as long as midwidth, lateral margin mid-half weakly convex, without setae, distal margin weakly concave, with long marginal cuticular scales, apex narrowly rounded; *stylet* in retracted position reaching apex. *Pleopod 3* endopod 2.5 midwidth; exopod article 1 2.6 as long as wide, not extending to endopod apex, lateral margin fringed with cuticular scale-spines; article 2 0.6 as long as article 1, lateral and mesial margins with spine-like cuticular scale-setae (mesial margin with setae).

*Uropod* peduncle extending slightly beyond margin of pleotelson, mediodistal corner strongly produced and acute, distolateral margin 3 simple submarginal setae, mesial margin finely serrate. Exopod 0.7 as wide as endopod, 1.3 as long as wide, with 7 simple setae. Endopod 1.2 as long as wide, 3.3 as long as peduncle proximolateral margin, apex with 0 long simple setae (with 7 pedunculate penicillate setae).

**Female.** *Pleopod 2* 1.2 as long as proximal width, lateral margins weakly convex, apex with 4 sub-apical simple setae.

#### Size.

Males 1.3–2.3 mm, mean 2.0 mm (*n*=6); ovigerous females 2.3–3.1 mm, mean 2.7 mm (*n*=3), non-ovigerous females 1.3–3.07 mm, mean 2.2 mm (*n*=11); all from type series.

#### Colour pattern.

Head with long dark-brown band extending to lateral margins, and occupying 76% of head length; anterior margin of colour band weakly indented; all other somites white or clear.

#### Variation.

Pleotelson with six acute teeth (serrations) on lateral margin; anterior most tooth sometimes small and inconspicuous.

#### Remarks.

*Joeropsis makrogenys* sp. n. is characterised by a brown head band occupying more than 70% of the head length; short, sub-quadrate, anteriorly concave pseudorostrum; antennula article 1 distolateral angle strongly serrate, large and asymmetrical mandible incisors, and the maxilliped endite with a smooth and obliquely angled distomesial margin, lacking a distinct mesial notch. The mandible and spine row morphology is unique within the genus, and the broadly rounded posterior incisory cusp of the right mandible can usually be seen under stereomicroscopy, in both adults and juveniles.

The colour pattern and mandibular morphology is unique, but is similar to that of *Joeropsis indica* Müller, 1991b. Comparing the type specimens of *Joeropsis indica* to *Joeropsis makrogenys* sp. n. shows that the left mandible incisor of *Joeropsis indica* is similar to that of *Joeropsis makrogenys*, but the right mandible of *Joeropsis indica* lacks the broad cusp; the maxilliped endite of *Joeropsis indica* (Fig. 12B) distal margin is serrated but not oblique and, contrary to Müller’s (1991b) description, does have distolateral tubercular spines; antenna 1 article 2 is clearly not serrated and the uropods have a weak terminal spike.

The similar *Joeropsis sandybrucei* Bruce, 2009 differs from *Joeropsis makrogenys* sp. n. in having the distolateral margin of antennula article 1 smooth (vs serrate), shorter pseudorostrum with mesially angled lateral margins, the maxilliped endite distal margin not oblique, and both mandible incisors have symmetrical acute cusps. The misidentified non-type material of *Joeropsis sandybrucei* is here re-identified; *Joeropsis sandybrucei* has not been collected at Lizard Island.

**Figure 11.**
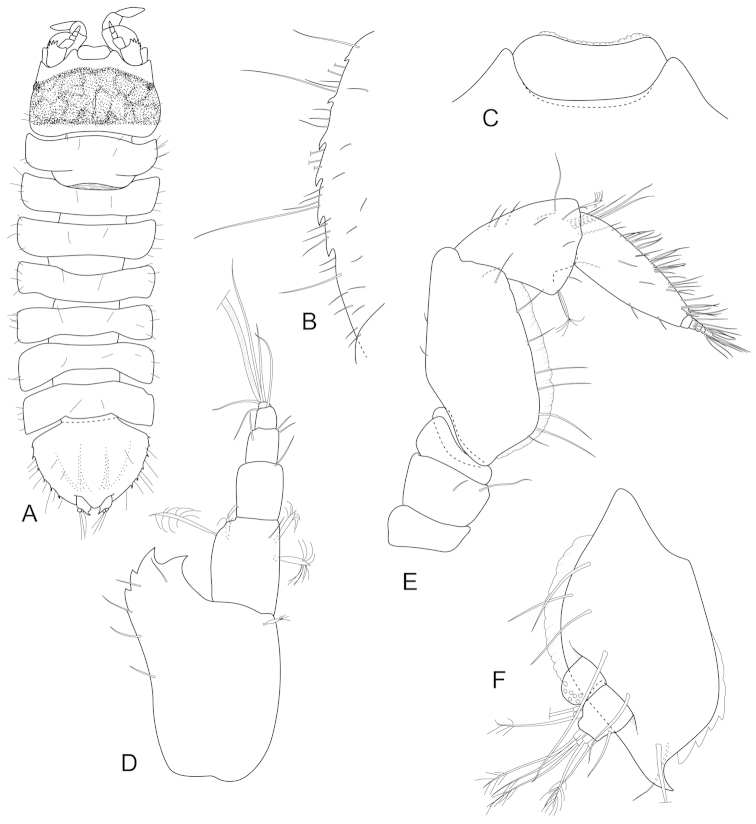
*Joeropsis makrogenys* sp. n. **A, C** holotype; remainder male paratype (2.3. mm) MTQ W34010. **A** dorsal view **B** pleotelson lateral margin **C** pseudorostrum **D** antenna 1 **E** antenna 2 **F** uropod.

**Figure 12.**
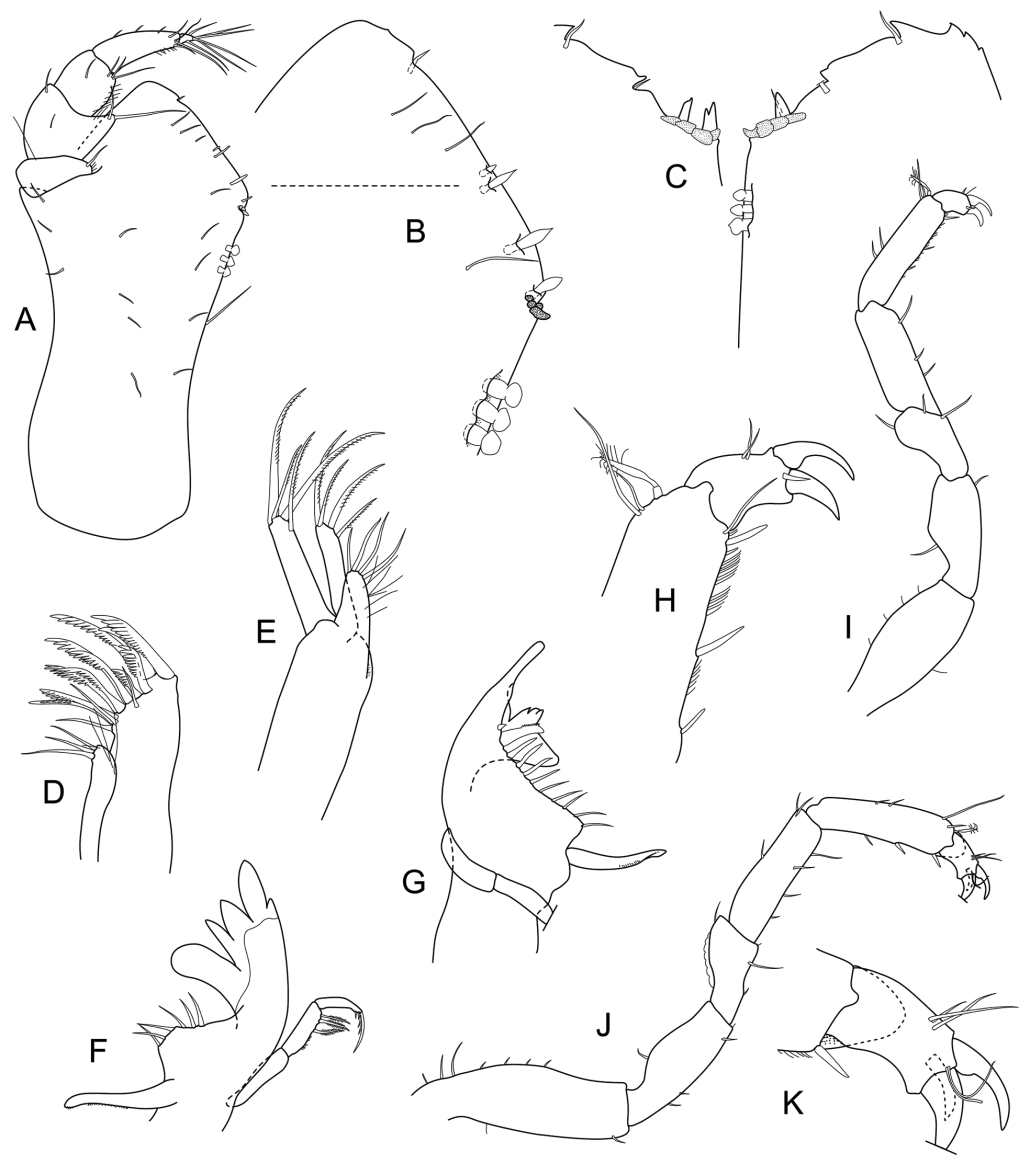
*Joeropsis makrogenys* sp. n. Male paratype (2.3 mm) MTQ W34010, except **C. A** maxilliped **B** maxilliped endite, distomesial margin **C** *Joeropsis indica* Müller, 1991: distomesial margins left and right maxilliped endites **D** maxillula **E** maxilla **F** right mandible **G** left mandible **H** pereopod 1 dactylus **I** pereopod 1 **J** pereopod 7 **K** pereopod 7 dactylus.

**Figure 13.**
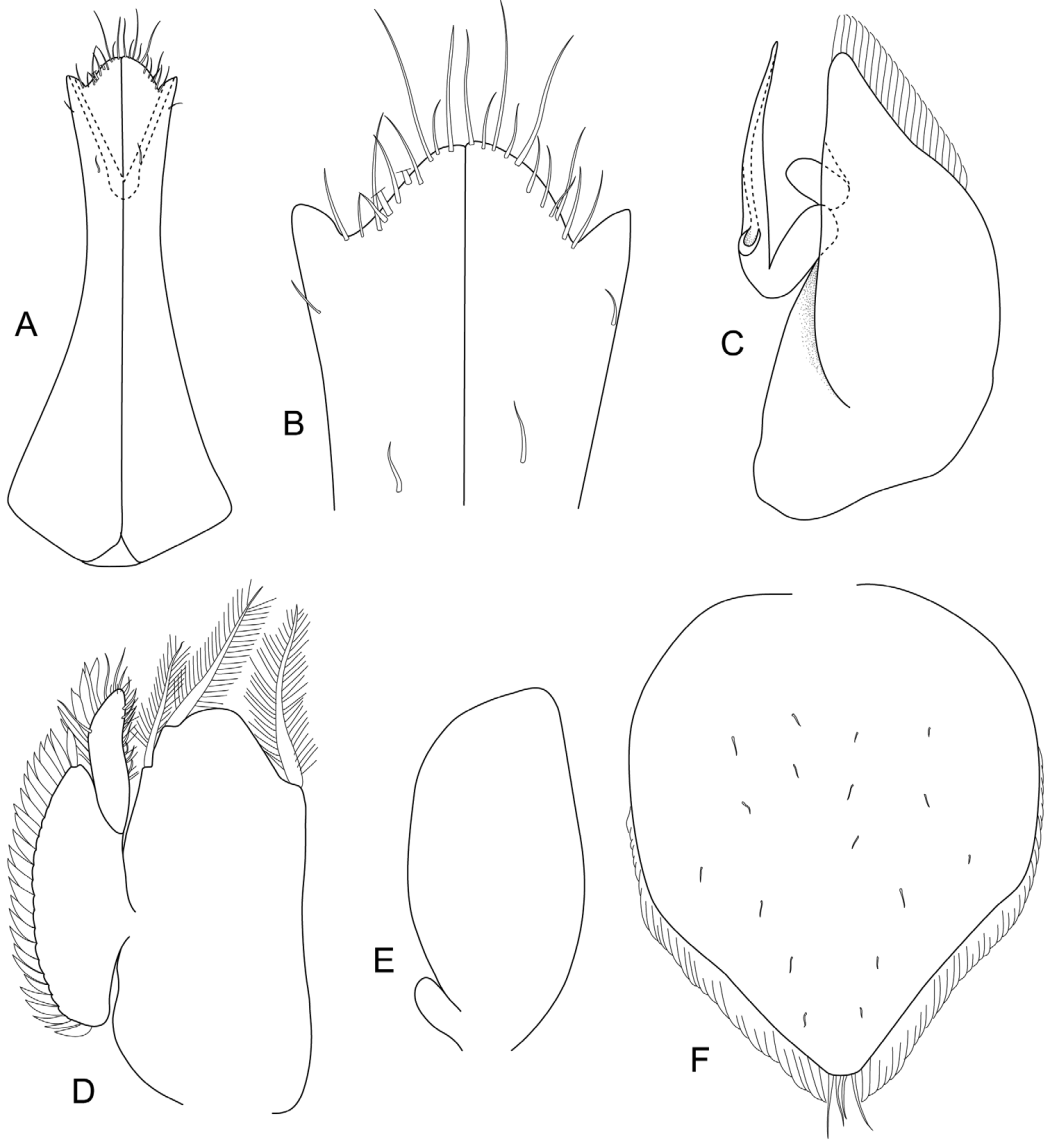
*Joeropsis makrogenys* sp. n. **A–E** male paratype (2.3 mm) MTQ W34010. **A** pleopod 1 **B** pleopod 1, apex **C** pleopod 2 **D** pleopod 3 **E** pleopod 4 **F** female paratype MTQ W34010 pleopod 2.

#### Distribution.

Lizard Island and nearby islands and reefs, South Direction Island, Hicks, Day and Yonge Reefs (Fig. 1); intertidal to 14 metres, with one sample from 30 metres; also Madang, Papua New Guinea.

#### Etymology.

The epithet combines the Greek words makros (long) and genys (jaw) alluding to the long cusp on the mandible incisor.

### Joeropsis mije sp. n.

Taxon classificationAnimaliaIsopodaJoeropsididae

http://zoobank.org/61FE99A9-D559-4F88-A7B0-69C197A97CE4

Figs 14 , 15 , 16

#### Material.

All material from the Lizard Island region, including outer barrier reefs.

*Holotype*. ♂ (1.8 [part dissected], 1.4 mm), Yonge Reef, 14.60681°S, 145.6311°E, 20 February 2009, outer reef front, dead coral, 30 m, stn LIZ09-15B, coll. S. Smith & JC (MTQ W31908, including dissection slide).

*Paratypes*. ♀ (ovig 1.4 mm [dissected]), Hicks Reef, 14.44803°S, 145.4992°E, 21 February 2009, outer reef front, small coral heads and rubble, 15 m, coll. MB-P & NLB, stn LIZ09-16C coll. NLB & MB-P (MTQ W31911). ♀ (non-ovig 1.1 mm), Hicks Reef, 14.44803°S, 145.4992°E, 21 February 2009, outer reef front, dead coral heads on reef edge, 5–7 m, LIZ09-16E, coll. NLB & MB-P (MTQ W31909). 2 ♂ (1.2, 1.0 mm), Day Reef, 14.48356°S, 145.5459°E, 13 February 2009, outer reef, fine rubble, 10 m, stn LIZ09-04C, coll. MB-P (MTQ W31910). ♂ (1.1 mm, poor condition), Day Reef, 14.48356°S, 145.5459°E, 13 February 2009, outer reef, coral heads in gully, 10 m, LIZ09-04B, coll. MB-P (MTQ W31906). ♀ (ovig. 1.3 mm), Day Reef, 14.47119°S, 145.5297 °E, 13 February 2009, outer reef, coral rubble in cave on vertical wall, 10–12 m, stn LIZ09-03B, coll. MB-P (MTQ W31907). ♂ (1.1 mm), Day Reef, 14.48283°S, 145.5564°E, 19 February 2009, outer reef front, small rubble in gully, 7.5 m, stn LIZ09-13A, coll. MB-P & NLB (MTQ W31284). ♂ (1.6 mm), Yonge Reef, 14.61383°S, 145.6182°E, 18 February 2009, back reef, small coral rubble on sand, 15 m, stn LIZ09-10F, coll. MB-P & NLB (MTQ W31283; microscope slide). ♂ (1.0 mm), ♀ (non-ovig 1.2 mm), Hicks Reef, 14.44803°S, 145.4992°E, 21 February 2009, outer reef front, coral rubble under bommie overhang, 15 m, stn LIZ09-16B, coll. NLB & MB-P (MTQ W31912).

*Also examined*. *Holotype* of *Joeropsis minuta* Müller, 1989. ♂ (1.0 mm), Französisch Polynesien, Iles du Vent, Mooréa. Temae, Islet Riff, NE vom Flughafen, tote Korallen nahe Strand, 2 m, Tiefe, tote Korallen, 31 March 1988, coll. H.-G. Müller (SMF 17689); 6 ♂ and ♀ paratype dissection, 6 microslides (SMF 17692). Near of airport, Tema’e, Mooréa, French Polynesia, *c.* 17.49°S, 149.76°W.

#### Description.

*Body* 2.9 as long as greatest width, dorsal surfaces smooth, with few setae. *Cephalon* length 0.6 width, lateral margins weakly concave, finely serrate (at mid-length). *Pseudorostrum* 1.5 as long as proximal width (with longitudinal ridge), anterior margin acute. *Eyes* lateral, with 12 ommatidia, colour orange. *Pereonites* not compact, widely spaced, without dorsal carinae; *tergite lateral margin* truncate, lateral margins smooth; median keels not observed. *Pleotelson* width 1.4 length; dorsal surface with weak and indistinct sub-lateral ridge, caudomedial lobe broadly rounded, not extending posteriorly beyond uropods; lateral margins weakly convex, each with 6–7 spines.

*Antenna 1* with 5 articles; article 1 1.0 as long as wide, distolateral angle not lobed, strongly serrated, distomesial margin weakly serrate; article 2 0.8 as long as article 1, 1.3 as long as wide; lateral margins of articles 1 and 2 with weak cuticular scales on distal margins; article 3 0.2 as long as article 2; article 4 1.7 as long as article 3 (articles 3 and 4 distal angles acute); article 5 2.7 as long as article 3, 1.9 as long as proximal width. *Antenna 2* peduncle article 5 1.8 as long as articles 1–4 combined, 4.8 as long as article 3, 1.2 as long as wide, lateral margin strongly convex, with prominent cuticular scales (and evenly spaced marginal setae), mesial margin convex; article 6 1.4 as long as width, distally weakly expanded, distal width 1.6 proximal width, 0.5 as long as article 5, lateral margin without cuticular scales, mesial margin with 12 simple setae, distodorsal surface with scattered simple setae; flagellum with 5 articles, article 1 0.8 as long as peduncle article 6, 2.1 as long as combined lengths of remaining articles.

*Mandible palp* article 2 with 2 long biserrate setae, article 3 with 3 long pectinate setae. Right incisor with symmetrical cusps, margins convex, distally acute. *Molar process* distal quarter finely serrate. Right mandible spine row composed of 9 spines. *Maxilla 1* lateral lobe with 12 strongly serrate RS, and 2 simple RS; mesial lobe with 4 long, simple RS. *Maxilla 2* lateral lobe with 4 long, curved, finely serrate setae (2 short, 2 long); middle lobe with 4 long serrate setae (2 short, 2 long), mesial lobe with 4 long simple setae and many long setules. *Maxilliped endite* 2.3 as long as greatest width, extending beyond palp, distal margin subtruncate, with 4 prominent serrations, with shallow distomesial concavity, with 3 mesial tubercular RS (and laterally with 1 distal rounded and finely serrated RS), distomesial margin with 2 coupling setae. *Maxilliped palp* article 2 3.6 as long as article 1, mesial lobe extending to article 4, distomesial margin with 3 simple setae; article 3 0.3 as long as article 2 (with ventral lobe), distomesial margin with 1 simple seta; article 4 4.2 as long as wide, mesial margin straight, distally with 2 setae; article 5 0.1 as long as 4, with 2 terminal setae.

*Pereopod 1* ischium 2.3 as long as wide; merus 0.6 length of ischium, 1.7 as long as wide; carpus 0.8 as long as ischium, 2.2 as long as wide; propodus 3.8 as long as wide, superior margin 2 simple setae (and prominent penicillate seta at distal angle); inferior margin with 2 acute RS, dactylus 0.5 as long as propodus, with 2 claws. *Pereopods 2–7* sub-similar, more slender than pereopod 1, each with 3 claws. *Pereopod 7* ischium 2.4 as long as wide, superior margin weakly convex at midpoint, with 1 simple seta, inferior distal angle with 1 seta; merus 0.6 as long as ischium, 1.7 as long as wide, superodistal angle with 2 simple setae; carpus 0.8 as long as ischium, 2.1 as long as wide, inferior margin with 1 simple seta, superior distal angle with 2 prominent pappose setae; propodus 1.0 as long as ischium, 3.3 as long as wide, inferior margin with 3 acute RS, superior margin with 2 simple setae; dactylus 0.6 as long as propodus.

*Pleopod 1* 2.0 as long as greatest width, lateral margin strongly concave with mid-length straight, apical lobe broadly rounded, with short marginal setae, lateral margin with slender setae, distolateral lobe blunt, not extending to distal margin. *Pleopod 2* protopod 2.3 as long as midwidth, lateral margin mid-half weakly convex, without setae (with fine cuticular scales), distal margin straight, without marginal cuticular scales (with setae), apex acute; *stylet* in retracted position extending beyond apex. *Pleopod 3* endopod 2.3 midwidth (medially fused to protopod); exopod article 1 3.5 as long as wide, extending beyond apex, lateral margin densely fringed with cuticular scale-setae; article 2 0.5 as long as article 1, lateral and mesial margins with short cuticular scale-setae (mesial margin with 3 setae).

*Uropod* peduncle extending well beyond margin of pleotelson, mediodistal corner strongly produced and acute, distolateral margin with 4 simple submarginal setae, mesial margin serrate. Exopod 0.5 as wide as endopod, 0.9 as long as wide, with 4 simple setae. Endopod 1.0 as long as wide, 0.8 as long as peduncle proximolateral margin, apex with 3 long simple setae.

**Female.** *Pleopod 2* 1.3 as long as proximal width, lateral margins weakly convex, posterior margins straight, apex with 2 sub-apical simple setae (surface with sub-mesial row of setae). *Antenna 2* peduncle article 5 not broadly expanded.

#### Colour pattern.

Head with diffuse, low-density transverse dark-brown band across the anterior 50% of the head; dorsal surfaces otherwise with few and faint chromatophores, mostly on pereonites 5–7 and anterior of pleotelson.

#### Size.

Males 1.0–1.8 mm (average 1.2 mm, *n*=8); ovigerous females 1.3–1.4 mm and non-ovigerous females 1.1–1.2 mm.

#### Remarks.

*Joeropsis mije* sp. n. is the smallest of the currently known Australian species of this genus and is best identified by the small size, acute pseudorostrum, diffuse dark brown head band, head lateral margin with two to four serrations just posterior to the eye, and the presence of only a few chromatophores over the dorsum; males are distinctive in having antenna 2 peduncle article 5 with an almost circular outline, a character readily observed in dorsal view.

*Joeropsis minuta* Müller (1989, fig. 30) is closely similar to *Joeropsis mije* sp. n. but the latter can be distinguished by the following characters (state for *Joeropsis minuta* in parentheses): head lateral margin weakly convex (mid-lateral margin weakly concave), pseudorostrum 2.5 as long that wide (as long as wide), pereonites widely spaced with lateral margins not in contact (closely spaced, pereonites 1–4 overlapping slightly) and pleotelson caudomedial lobe not extending posteriorly beyond uropods (extending beyond uropods).

**Figure 14.**
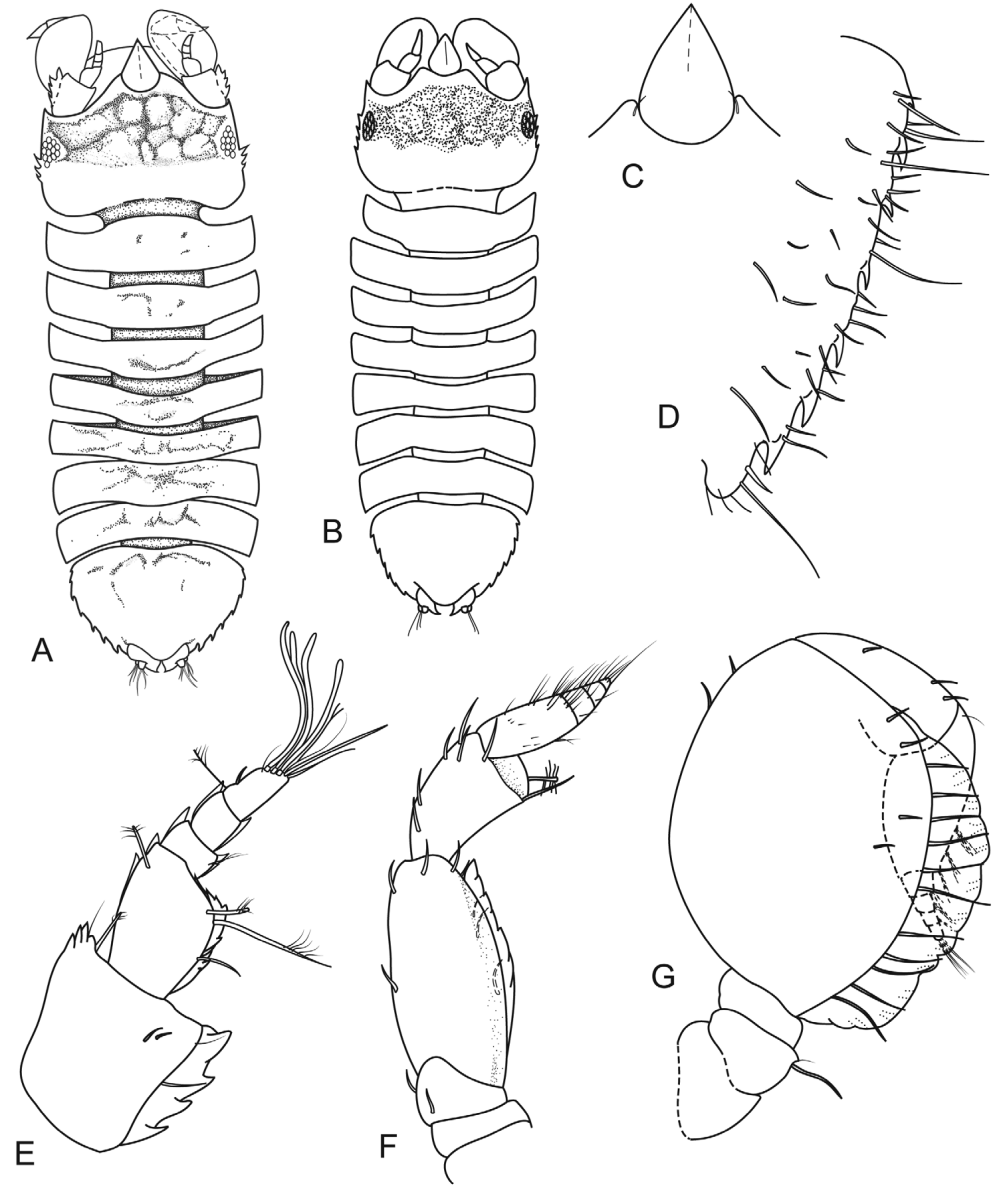
*Joeropsis mije* sp. n. **A, C** holotype (1.8 mm MTQ W31908) **E, F** female (ovig 1.4 mm MTQ W31911) **B, D, G** male (1.6. mm MTQ W31283). **A**, dorsal view **B** dorsal view, immature male **C** pseudorostrum **D**, pleotelson lateral margin **E** antenna 1 **F** antenna 2 **G** male antenna 2.

**Figure 15.**
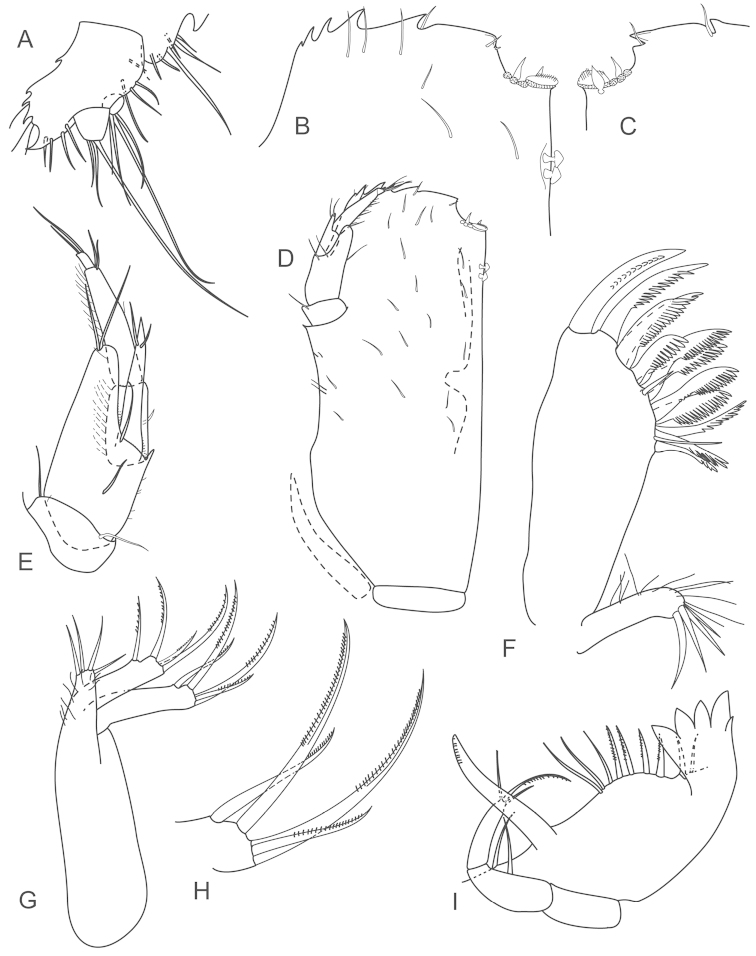
*Joeropsis mije* sp. n. **A, I** male (1.6 mm MTQ W31283); **B–H** male (1.8 mm MTQ W31908). **A** uropod **B** right maxilliped, distal margin **C** left maxilliped, distomesial margin **D** maxilliped **E** maxilliped palp **F** maxillula **G** maxilla **H** maxilla, mesial lobe setae **I** mandible.

**Figure 16.**
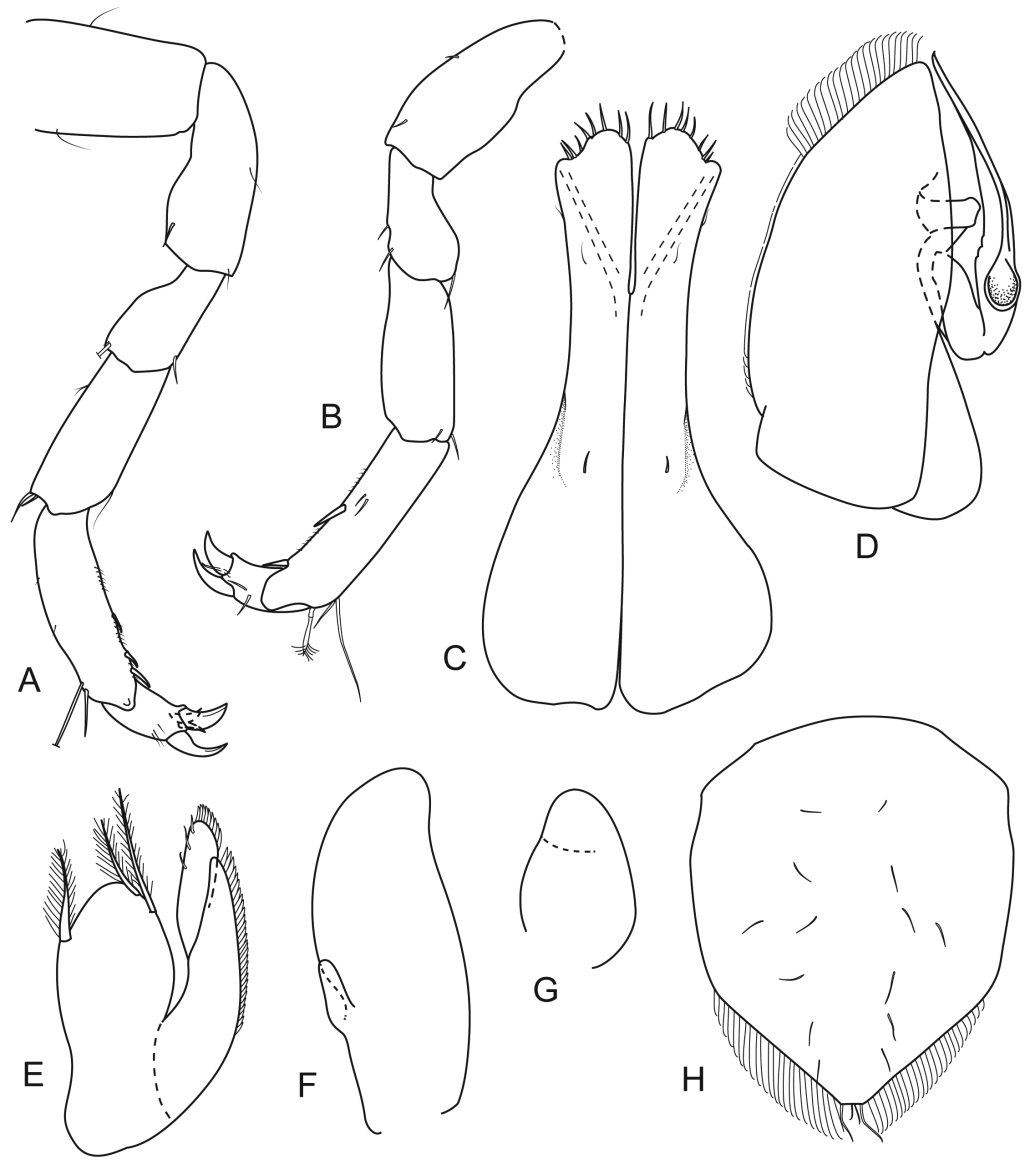
*Joeropsis mije* sp. n. **A–D** male (1.6. mm MTQ W31911) **E–H** female (1.6 mm) MTQ W30468 **A** pereopod 7 **B** pereopod 1 **C–G** pleopods 1–5 respectively **H** pleopod 2.

#### Distribution.

Hicks, Day and Yonge Reefs (Fig. 1); depths 10 to 15 metres.

#### Etymology.

The epithet is an Aboriginal word meaning little, alluding to the small size of this species; noun in apposition.

### Joeropsis panstikta sp. n.

Taxon classificationAnimaliaIsopodaJoeropsididae

http://zoobank.org/A1F931ED-3628-4786-AF77-BAE879DE4C5C

Figs 17 , 18 , 19

#### Material.

*Holotype*. ♂ (3.4 mm), Yonge Reef, 14.60681°S, 145.6311°E, 20 February 2009, outer reef front, dead coral, 30 m, stn LIZ09-15B, coll. S. Smith & JC (MTQ W31276).

*Paratypes*. 7 ♂ (3.3, 3.3, 3.2 [dissected], 3.1 [dissected], 2.8 2.3, 2.1 mm), ♀ 4 (ovig. 3.2 [dissected], non-ovig. 2.9, 2.8, 2.1 mm) same data as holotype (MTQ W31277). 4 ♀ (ovig. 3.0), Yonge Reef, 14.57972°S, 145.61010°E, 20 April 2008, passage, dead *Acropora* head, 17 m, stn CGLI-046A, coll. MB-P (MTQ W13980). ♂ (2.4 mm), 2 ♀ (non-ovig. 3.4, 3.0 mm), Yonge Reef, 14.61383 °S, 145.6182°E, 18 February 2009, back reef, small coral rubble on sand, 15 m, stn LIZ09-10F, coll. MB-P & NLB(MTQ W31278). ♀ (non-ovig. 3.4 mm), Hicks Reef, 14.44803°S, 145.4992°E, 21 February 2009, outer reef front, dead coral heads on reef edge, 5–7 m, LIZ09-16E, coll. NLB & MB-P (MTQ W31279). 3 ♂ (3.3, 2.5, 2.3 mm), Hicks Reef, 14.44803°S, 145.4992°E, 21 February 2009, outer reef front, small rubble on sand, base of reef edge, 8 m, stn LIZ09-16F, coll. NLB & MB-P (MTQ W31280). ♂ (3.3 mm), Day Reef, 14.47503°S, 145.5366°E, 22 February 2009, outer reef edge, coral rubble, 30 m, stn CWLI-050, coll. S. Smith (MTQ W31281). 16, not measured (♂ 3.2 mm [dissected]), Day Reef, 14.48356°S, 145.5459°E, 13 February 2009, outer reef, coral heads in gully, 10 m, LIZ09 04B, coll. MB-P (MTQ W31195).

*Additional material*. ♂ (2.7 mm), ♀ (ovig. 3.2, non-ovig. 3.3 mm), Day Reef, 14.50239°S, 145.5089°E, 21 February 2009, outer reef slope, large coral rubble, 30 m, stn CWLI048, coll. JC & K. Mills (MTQ W34165). 9, not measured, Yonge Reef, 14.62317°S, 145.6201°E, 13 February 2009, reef pass, dead coral heads, 5 m, stn LIZ09-11A, coll. NLB & MB-P (MTQ W31218). 1, not measured, Day Reef, 14.47119 °S, 145.5297 °E, 13 February 2009, outer reef, dead coral on vertical wall, 10–12 m, stn LIZ09-03A, coll. MB-P (MTQ W30465). 7, not measured, Day Reef, 14.47119 °S, 145.5297 °E, 13 February 2009, outer reef, coral rubble in cave on vertical wall, 10–12 m, stn LIZ09-03B, coll. MB-P (MTQ W30466). 7, not measured, Day Reef, 14.50525°S, 145.5612°E, 22 February 2009, outer reef front. coral rubble, 27–29 m, stn LIZ09-17A, coll. Shawn Smith & JC (MTQ W31103). 1, not measured, Hicks Reef, 14.44803°S, 145.4992°E, 21 February 2009, outer reef front, dead coral heads on spur, 16 m, stn LIZ09-16D, coll. MB-P & NLB (MTQ W31287). ♂ (3.0 mm), Day Reef, 14.48539°S, 145.5464°E, 19 February 2009, outer reef front, coral rubble in gully, 17 m, stn LIZ09-12F, coll. NLB & MB-P (MTQ W31081). ♂, ♀ and half specimen (unmeasured) Hicks Reef, 14.48051°S, 145.4873°E, 14 February 2009, outer reef, coral rubble, 2–18 m, stn CWLI-020, coll. C. Watson & Kade Mills (MTQ W31382). ♂ (1.7 mm), Day Reef, 14.48356°S, 145.5459°E, 13 February 2009, outer reef, fine rubble, 10 m, stn LIZ09-04C, coll. MB-P (MTQ W31700). Juvenile, off Coconut Beach, Lizard Island, 14.68441°S, 145.47197°E, 17 February 2009, reef front., small rubble on sand between bommies, 4.5 m, stn LIZ09-09C, coll. NLB & MB-P (MTQ W31285).

#### Description.

*Body* 3.5 as long as greatest width, dorsal surfaces smooth, with few setae. *Cephalon* length 0.7 width, lateral margins weakly concave, smooth. *Pseudorostrum* 0.6 as long as proximal width, anterior margin rounded. *Eyes* sublateral, with 16 ommatidia, colour dark brown. *Pereonites* not compact, widely spaced, without dorsal carinae; *tergite lateral margin* subtruncate, lateral margins smooth; median keels on sternites 4–7, keels well developed. *Pleotelson* width 1.2 length; dorsal surface with weak and indistinct sub-lateral ridges, caudomedial lobe narrowly rounded; lateral margins weakly convex, each with 1–2 spines.

*Antenna 1* with 7 articles; article 1 1.4 as long as wide, distolateral angle weakly lobed, not serrated, distomesial margin weakly serrate; article 2 0.6 as long as article 1, 1.4 as long as wide; lateral margins of articles 1 and 2 without cuticular scales; article 3 0.4 as long as article 2; article 4 0.9 as long as article 3; article 5 0.8 as long as article 3, 2.6 as long as proximal width, distally with 1 aesthetasc (further 2 short articles present). *Antenna 2* peduncle article 5 1.6 as long as articles 1–4 combined, 3.8 as long as article 3, 1.6 as long as wide, lateral margin convex, with prominent cuticular scales, mesial margin straight; article 6 2.1 as long as width, distally weakly expanded, distal width 1.9 proximal width, 0.7 as long as article 5, lateral margin with cuticular scales on distal one-third, mesial margin with 8 simple setae, distodorsal surface with few simple setae; flagellum with 8 articles, article 1 1.1 as long as peduncle article 6, 1.9 as long as combined lengths of remaining articles.

*Mandible palp* article 2 with 3 long biserrate setae (terminally spatulate), article 3 with 9 long pectinate setae. Right incisor with symmetrical cusps, margins convex, distally acute; left mandible incisor similar to right incisor. *Molar process* distal half finely serrate. Right mandible spine row composed of 8 spines; divided by truncate lobe; left spine row without lacinoid spine. *Maxilla* 1 lateral lobe with 11 strongly serrate RS, and 2 simple RS; mesial lobe with 3 long, simple RS. *Maxilla* 2 lateral lobe with 4 long, curved, finely serrate setae; middle lobe with 4 long serrate setae, mesial lobe with 4 long simple setae and many long setules. *Maxilliped endite* 1.9 as long as greatest width, extending to distal margin of palp article 4, distal margin evenly rounded, mesially with 2 large serrations, with shallow distomesial concavity, with 4 mesial tubercular RS, distomesial margin with 3 coupling setae. *Maxilliped palp* article 2 3.7 as long as article 1, mesial lobe extending to distal margin of article 3, distomesial margin with 2 simple setae; article 3 0.5 as long as article 2, distomesial margin with 2 simple setae; article 4 4.0 as long as wide, mesial margin weakly concave, distally with 7 setae; article 5 0.2 as long as 4, with 4 terminal setae.

*Pereopod 1* basis 3.0 as long as wide, superior margin with 1 simple seta; ischium 0.7 as long as basis, 3.4 as long as wide; merus 0.6 length of ischium, 2.1 as long as wide; carpus about 1.0 (0.95)as long as ischium, 4.2 as long as wide; propodus 4.2 as long as wide, superior margin 4 simple setae (and 1 distal penicillate seta); inferior margin with 4 acute RS, dactylus 0.3 as long as propodus, with 2 claws. Pereopods 2–7 sub-similar, more slender than pereopod 1, each with 3 claws. *Pereopod 7* basis 2.9 as long as wide; superior margin with 3 short simple setae; ischium 0.7 as long as basis, 2.8 as long as wide, superior margin with 2 simple setae, inferior distal angle without setae; merus 0.6 as long as ischium, 1.5 as long as wide, superodistal angle with 1 simple seta; carpus 0.9 as long as ischium, 3.9 as long as wide, inferior margin with 1 seta (distal three-quarters with cuticular scale-setae), superior distal angle with 1 prominent pappose seta; propodus 1.0 as long as ischium, 5.0 as long as wide, inferior margin with 5 acute RS, superior margin with 2 simple setae (and distal penicillate seta); dactylus 0.4 as long as propodus.

*Pleopod 1* 2.4 as long as greatest width, lateral margin distally concave, apical lobe triangular, with long marginal setae, lateral margin stiff and slender setae, distolateral lobe acute, extending beyond distal margin. *Pleopod 2* protopod 2.4 as long as midwidth, lateral margin mid-half weakly convex, without setae, distal margin weakly concave, with long marginal cuticular scales, apex narrowly rounded; *stylet* in retracted position reaching apex. *Pleopod 3* endopod 1.3 midwidth (fused to protopod); exopod article 1 2.5 as long as wide, not extending to endopod apex, lateral margin weakly fringed with cuticular scale-setae; article 2 0.8 as long as article 1, lateral and mesial margins with long cuticular scale-setae (mesial only, lateral very weakly scaled).

*Uropod* peduncle extending slightly beyond margin of pleotelson, mediodistal corner strongly produced and acute, distolateral margin 3 simple submarginal setae, mesial margin serrate. Exopod 0.6 as wide as endopod, 1.4 as long as wide, with 9 simple setae (3 long). Endopod 1.3 as long as wide, 0.4 as long as peduncle proximolateral margin, apex with 9 long simple setae.

**Female.** *Pleopod 2* 1.3 as long as proximal width, lateral margins weakly convex, posterior margins sinuate (with cuticular scale-setae), apex with 2 sub-apical simple setae. Pleotelson lateral margins each with 7 teeth.

#### Colour pattern.

All pereonites with reddish-brown chromatophores; head with long and darker band of chromatophores occupying 77% of head length, anterior margin of head band weakly and evenly convex, posterior margin forming blunt triangle; pereonite 1 lateral margins clear, narrow marginal clear area on tergite lateral margin 2–4.

#### Size.

Males 2.1–3.4 mm (mean 2.9 mm, *n*=14); ovigerous females 3.0–3.2 mm (*n*=2), non-ovigerous females 2.1–3.4 mm (mean 2.9 mm, *n*=6).

#### Variation.

Male pleotelson margin with 1 or 2 teeth only; females and small males usually with 7 teeth on each margin, with the anterior 2 or 3 teeth smaller than the posterior 3 or 4 teeth.

#### Remarks.

*Joeropsis panstikta* sp. n. is common on the outer reefs of the northern Great Barrier Reef and is also one of the largest species present, reaching a size of 3.4 mm. The entire dorsum is covered by clustered reddish-brown chromatophores, with a darker brown head, and the colour pattern together with anteriorly rounded pseudorostrum and large size allows for identification of this species.

**Figure 17.**
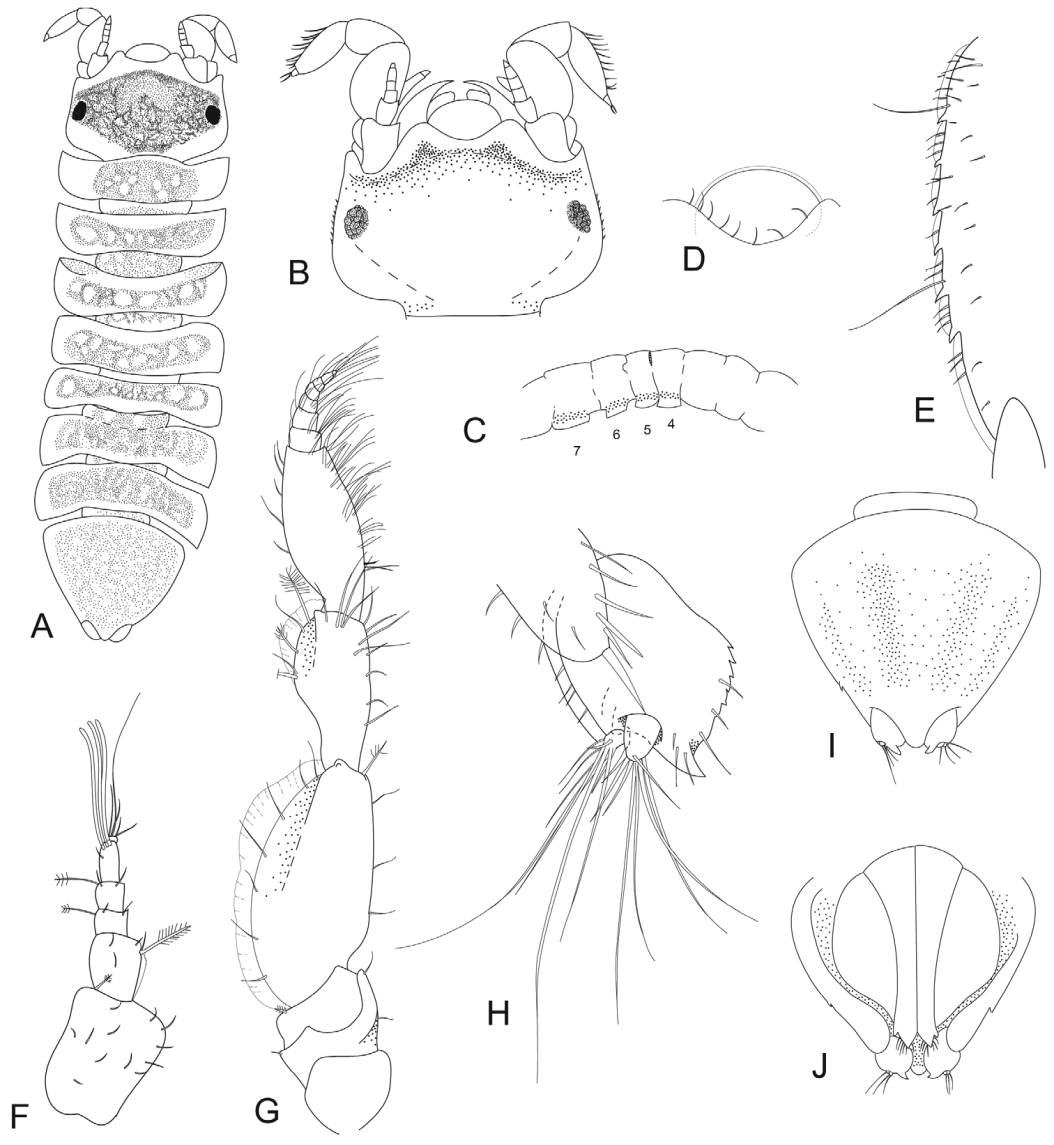
*Joeropsis panstikta* sp. n. **A–C, I, J** holotype; remainder male and female paratypes (3.1, 2.3 mm) MTQ W31277. **A** dorsal view **B** head, dorsal view **C** sternal carinae (sternites numbered) **D** pseudorostrum **E** female, pleotelson lateral margin **F** antenna 1 **G** antenna 2 **H** uropod **I** pleotelson, dorsal view **J** pleon, ventral view.

**Figure 18.**
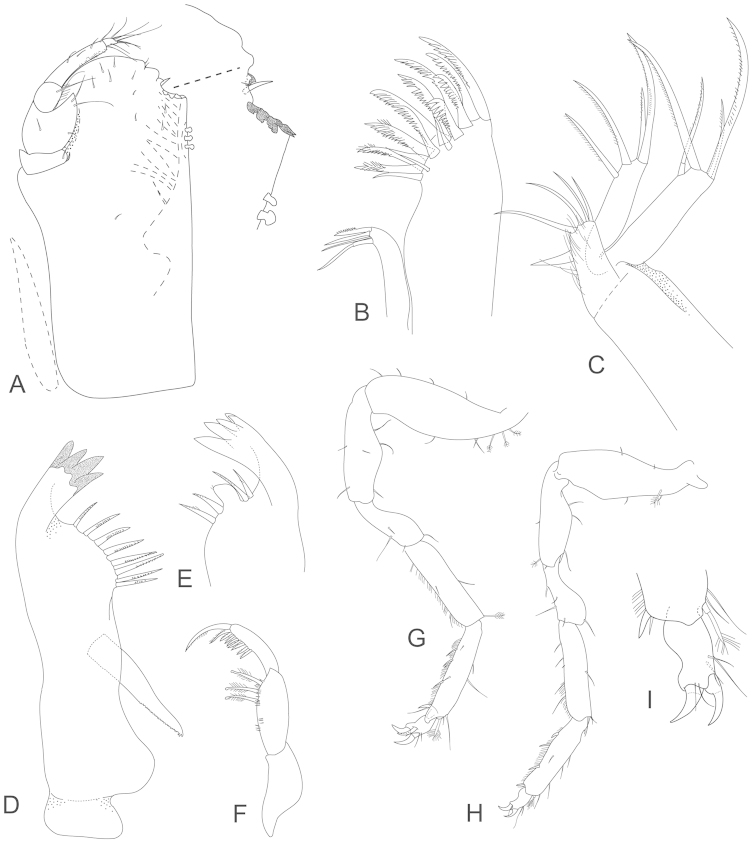
*Joeropsis panstikta* sp. n. Male paratype (3.1 mm) MTQ W31277. **A** maxilliped **B** maxilla **C** maxillula **D** right mandible **E** left mandible **F** mandible palp **G** pereopod 7 **H** pereopod 1 **I** pereopod 1 dactylus.

**Figure 19.**
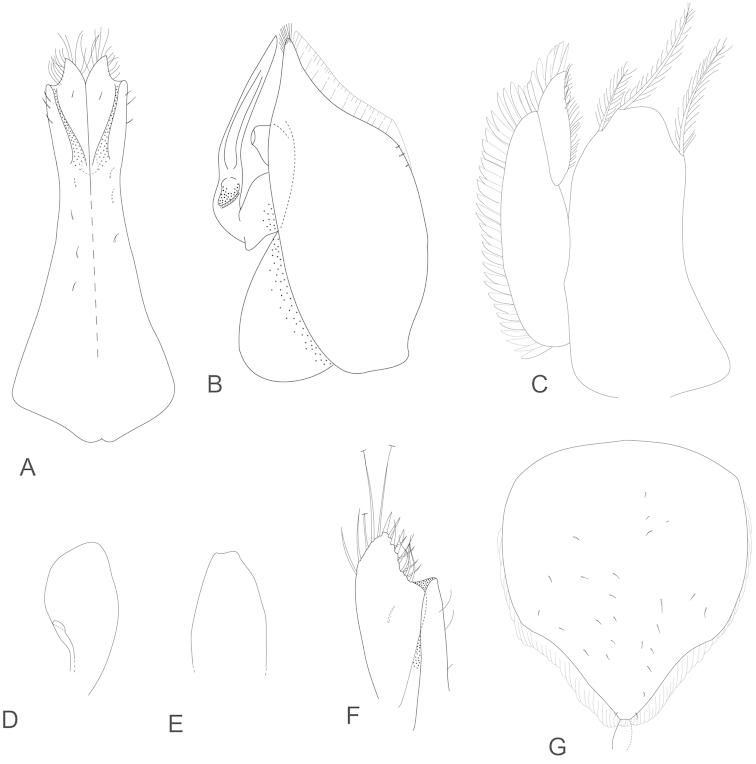
*Joeropsis panstikta* sp. n. **A–C, F** male paratype (3.1 mm) **D, F, G** female (3.0 mm) paratype; both MTQ W31277. **A–E** pleopods 1–5 respectively **F** pleopod 2 apex **G** female pleopod 2.

#### Distribution.

Frequent at Hicks, Day and Yonge Reefs; once only at Coconut Beach, Lizard Island (Fig. 1); depths 5 to 30 metres.

#### Etymology.

The epithet is a contraction of the Greek ‘pantostikta’ (παντός+στικτο), meaning ‘spotted all over’.

### Joeropsis specca sp. n.

Taxon classificationAnimaliaIsopodaJoeropsididae

http://zoobank.org/AE88E898-9E84-4D14-8005-1DAAB7E6E4C4

Figs 20 , 21 , 22

#### Material.

*Holotype*. ♂ (2.2 mm), Martin Reef, 14.75600°S, 145.36271°E, 30 August 2010, reef flat, dead corals, 5 m, LI10-35, coll. J. Reimer (MTQ W33804).

*Paratypes*. 5 ♂ (2.8, 2.6 [broken; dissected], 2.0, 1.9, 1.8 mm), 7 ♀ (ovig 2.7, 2.0 mm, non-ovig. 2.7, 2.5[dissected], 2.4, 1.9, 1.8 mm), same data as holotype (MTQ W32664).

#### Description.

*Body* 4.0 as long as greatest width, dorsal surfaces finely granular, with few setae. *Cephalon* length 0.6 width, lateral margins weakly sinuate, smooth. *Pseudorostrum* 0.7 as long as proximal width, anterior margin narrowly truncate, apex narrowly excavate. *Eyes* lateral, with 12–14 ommatidia, colour dark brown. *Pereonites* compact, close to each other, without dorsal carinae (with low tubercle, visible only with relief lighting); *tergite lateral margin* rounded, lateral margins smooth; median keels not observed. *Pleotelson* width 1.0 length, dorsal surface with weak and indistinct sub-lateral ridges, caudomedial lobe sub-acute; lateral margins convex, each with 6–7 spines.

*Antenna 1* with 5 articles (may be a 6th fused terminal article); article 1 1.3 as long as wide, distolateral angle weakly lobed, with serrations, distomesial margin not serrate; article 2 0.7 as long as article 1, 1.5 as long as wide; lateral margins of articles 1 and 2 with weak cuticular scales on distal margin of article 2; article 3 0.4 as long as article 2; article 4 0.7 as long as article 3; article 5 1.3 as long as article 3, 1.7 as long as proximal width, distally with 3 aesthetascs. *Antenna 2* peduncle article 5 1.6 as long as articles 1–4 combined, 1.8 as long as wide, lateral margin weakly convex, with prominent cuticular scales, mesial margin straight; article 6 1.8 as long as width, distally not expanded, distal width 1.3 proximal width, 0.8 as long as article 5, lateral margin without cuticular scales, mesial margin with 4 simple setae, distodorsal surface with few simple setae; flagellum with 8 articles, article 1 1.2 as long as peduncle article 6, 1.9 as long as combined lengths of remaining articles.

*Mandible palp* article 2 with 3 long biserrate setae (terminally spatulate), article 3 with 8 long pectinate setae. Right incisor with symmetrical cusps, margins convex, distally acute; left mandible incisor similar to right incisor. *Molar process* distal third finely serrate. Right mandible spine row composed of 9 spines; left mandible spine row composed of 10 spines, divided by truncate lobe, without lacinoid spine. *Maxilla* 1 lateral lobe with 11 strongly serrate RS, and 2 simple RS; mesial lobe with 3 long, simple RS. *Maxilla* 2 lateral lobe with 4 long, curved, finely serrate setae; middle lobe with 4 long serrate setae, mesial lobe with 4 long simple setae and many long setules. *Maxilliped endite* 2.1 as long as greatest width, extending to distal margin of palp article 4, distal margin evenly rounded, with 4 prominent serrations, with shallow distomesial concavity, with 4 mesial tubercular RS, distomesial margin with 3–4 coupling setae. *Maxilliped palp* article 2 2.3 as long as article 1, mesial lobe extending to distal margin of article 3, distomesial margin with 1 simple seta; article 3 0.5 as long as article 2, distomesial margin with 2 simple setae (and mesial row of dense cuticular scale-setae); article 4 3.4 as long as wide, mesial margin weakly concave, distally with 4 setae; article 5 0.2 as long as 4, with 8 terminal setae. *Epipod* 2.7 as long as basal width, distally acute; 0.9 as long as palp, 0.5 as long as endite.

*Pereopod 1* basis 3.2 as long as wide, superior margin with 4 simple setae, inferior margin with 2 simple setae; ischium 0.7 as long as basis, 3.1 as long as wide, superior margin with 3 simple setae, inferior margin with 1 simple seta; merus 0.6 length of ischium, 1.9 as long as wide, superior margin with 1 simple setae, inferior margin with 1 simple seta; carpus 0.8 as long as ischium, 3.9 as long as wide, superior margin with 2 simple setae, inferior margin with 2 simple seta; propodus 3.7 as long as wide, superior margin 2 simple setae (and prominent penicillate seta at distal angle); inferior margin with 3 acute RS, dactylus 0.4 as long as propodus, with 2 claws. Pereopods 2–7 sub-similar, more slender than pereopod 1, each with 3 claws (mesial claw slender). *Pereopod 7* basis 314 as long as wide; superior margin with 4 short simple setae, inferior margin without setae; ischium 0.8 as long as basis, 3.3 as long as wide, superior margin convex, with 3 simple setae, inferior distal angle with 1 seta; merus 0.4 as long as ischium, 2.1 as long as wide, superodistal angle with 1 simple seta, inferior margin with 1 simple seta; carpus 0.8 as long as ischium, 4.9 as long as wide, inferior margin with 3 setae, superior distal angle without prominent pappose setae; propodus 0.8 as long as ischium, 5.2 as long as wide, inferior margin with 3 acute RS, superior margin with 4 simple setae and 1 distal pappose seta; dactylus 0.5 as long as propodus.

*Pleopod 1* 2.5 as long as greatest width, lateral margin weakly concave, apical lobe broadly rounded, with long marginal setae, lateral margin with slender setae, distolateral lobe acute, not extending to distal margin. *Pleopod 2* protopod 2.5 as long as midwidth, lateral margin mid-half weakly convex, with setae (short setae and cuticular scales), distal margin weakly concave, with long marginal cuticular scales, apex narrowly rounded; *stylet* in retracted position extending beyond apex. *Pleopod 3* endopod 2.7 midwidth; exopod article 1 3.1 as long as wide, extending to endopod apex, lateral margin fringed with cuticular scale-spines; article 2 0.4 as long as article 1, lateral and mesial margins with spine-like cuticular scale-setae (lateral only, mesial with 6 fine setae).

*Uropod* peduncle extending slightly beyond margin of pleotelson, mediodistal corner strongly produced and acute, apex with 4 simple setae, distolateral margin with 2 simple submarginal setae, mesial margin smooth. *Exopod* 0.6 as wide as endopod, 1.0 as long as wide, with 8 simple setae. *Endopod* 1.3 as long as wide, 0.3 as long as peduncle proximolateral margin, apex with 10 long simple setae (and 3 penicillate setae).

**Female.** *Pleopod 2* 1.3 as long as proximal width, lateral margins weakly convex, posterior margins weakly concave, apex with 2 sub-apical simple setae.

#### Colour pattern.

Head with relatively short diffuse, low-density, irregular transverse dark-brown band across the mid 30–48% of the head; dorsal surfaces otherwise with few and faint chromatophores on pereonites 1–4 and 6, 7 and pleotelson; pleonite 5 clear, head and pereonite 4 consistently darkest, pereonites 1 and 2 with chromatophores but variable and pereonites 6 and 7 consistently paler than anterior pereonites.

#### Size.

Males 1.8–2.8 mm (average 2.7 mm, *n*=6); ovigerous females 2.0 and 2.7 mm and non-ovigerous females 1.8–2.7 mm (average 2.3 mm, *n*=5).

#### Variation.

Male pleotelson margin (*n*=7) with 6 (21%) or 7 (64%) teeth, the largest male with 2 mid-marginal teeth only; females with 7 (100%, *n*=7) teeth on each margin. Colour varies from near black to pale brown.

#### Remarks.

*Joeropsis specca* sp. n. may be identified by the colour pattern, in conjunction with a pseudorostrum with straight lateral margins that converge mesially to a narrowly excavate apex, relatively large eyes, pleotelson margins each with 5 or 6 spines and the rounded tergite lateral margin.

There are many shallow-water species of *Joeropsis* that present an irregular pattern of chromatophores over most of the dorsum. In several such species pereonite 5 is without chromatophores: these species include, for example, *Joeropsis dimorpha* Kensley & Schotte, 2002 (Seychelles), *Joeropsis faurei* Müller, 1991a (Réunion Island), *Joeropsis gertrudae* Müller, 1989 (Mooréa), *Joeropsis trilabes* Kensley, 2003 (Easter Island) and *Joeropsis bicornis* Kensley, 2003 (Easter Island). The intensity (how dark) and density of the patterning does vary, and the arrangement of the chromatophores may not allow for species separation on its own. Critical characters to reference in separating these species are morphological (see ‘Species recognition’, p. MS 9), in particular the shape of the pseudorostrum, details of the maxilliped, antennula and antenna, and the serration of the lateral margins of both the head and pleotelson.

**Figure 20.**
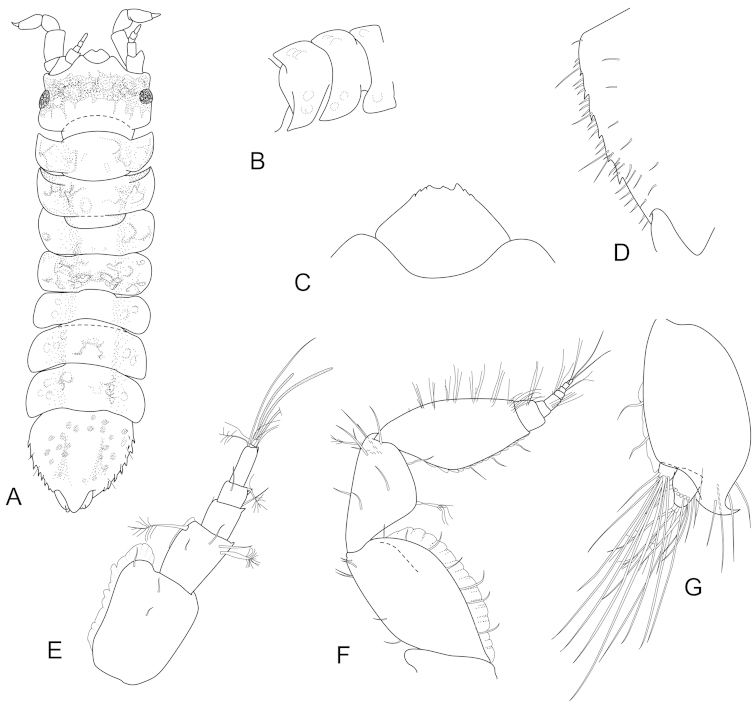
*Joeropsis specca* sp. n. **A, B** holotype **C–G** male paratype (2.6 mm) MTQ W32664. **A** dorsal view **B** oblique view, pereonites 5–7 **C** pseudorostrum **D** pleotelson lateral margin **E** antenna 1 **F** antenna 2 **G** uropod.

**Figure 21.**
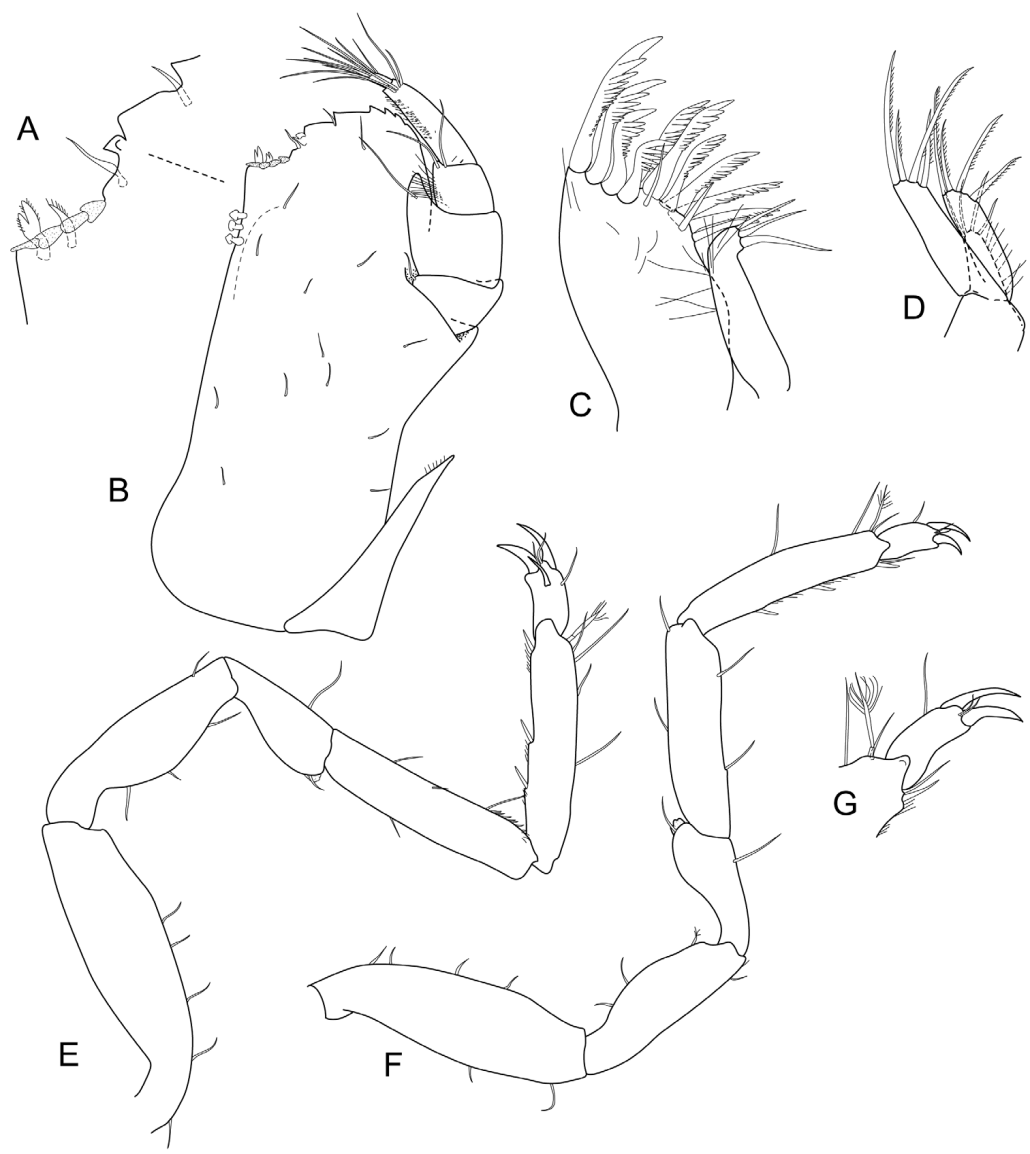
*Joeropsis specca* sp. n. All male paratype (2.6 mm) MTQ W32664. **A** maxilliped endite distomesial margin **B** maxilliped **C** maxillula **D** maxilla **E** pereopod 7 **F** pereopod 1 **G** pereopod 7 dactylus.

**Figure 22.**
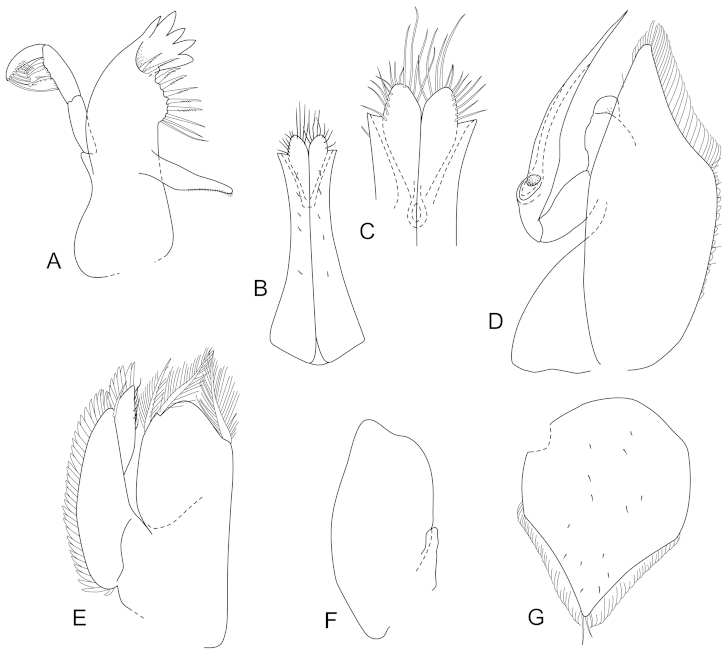
*Joeropsis specca* sp. n. All MTQ W32664 **A–F** male paratype (2.6 mm). **A** left mandible **B** pleopod 1 **C** pleopod 1 apex **D** pleopod 2 **E** pleopod 3 **F** pleopod 4 **G** female (2.5 mm) pleopod 2.

#### Distribution.

Known only from the type locality, Martin Reef (midway between Lizard Island and the mainland, Fig. 1); at a depth of 5 metres.

#### Etymology.

The epithet is the Anglo-Saxon *specca* meaning speckled.

### Joeropsis tropida sp. n.

Taxon classificationAnimaliaIsopodaJoeropsididae

http://zoobank.org/F3D029AC-3C68-4A72-AEA9-B8E7FD3679DC

Figs 23 , 24

#### Material.

*Holotype*. ♀ (non-ovig. 2.4 mm), Hicks Reef, 14.44803°S, 145.4992°E, 21 February 2009, outer reef front, *Halimeda* sand in groove, 10–12 m, stn LIZ09-16A, coll. NLB & MB-P (MTQ W33713).

*Paratypes*. 2 ♀ (ovig. 2.0 [dissected], non-ovig. 1.8 mm), same data as holotype (MTQ W33714).

#### Description.

*Body* 3.7 as long as greatest width, dorsal surfaces polished in appearance, without setae. *Cephalon* length 0.6 width, lateral margins weakly concave, smooth. *Pseudorostrum* 1.2 as long as proximal width, anterior margin acute (lateral margins weakly concave). *Eyes* lateral, with 12 ommatidia, colour dark brown. *Pereonites* compact, close to each other (posteriorly), 1–7 with paired sub-median carinae; *tergite lateral margin* subtruncate, lateral margins smooth; median keels on sternites 2–7, keels well developed (on sternites 2–4 and 6 and 7). *Pleotelson* width 1.1 length, dorsal surface with prominent rounded median ridge and paired submedian depressions, caudomedial lobe broadly rounded; lateral margins convex, each with 5–7 spines.

*Antenna 1* with 7 articles; article 1 1.4 as long as wide, distolateral angle not lobed, with cuticular scales, distomesial margin not serrate; article 2 0.6 as long as article 1, 1.5 as long as wide; lateral margins of articles 2 with prominent cuticular scales; article 3 0.1 as long as article 2; article 4 2.6 as long as article 3; article 5 2.5 as long as article 3, 1.5 as long as proximal width, distally with 3 aesthetascs. *Antenna 2* peduncle article 5 2.0 as long as articles 1–4 combined, 5.1 as long as article 3, 1.9 as long as wide, lateral margin convex, with small cuticular scales, mesial margin weakly convex; article 6 2 as long as width, distally expanded, distal width 1.7 proximal width, 0.6 as long as article 5, lateral margin without cuticular scales, mesial margin with 11 simple setae, distodorsal surface with scattered simple setae; flagellum with 4 articles, article 1 0.7 as long as peduncle article 6, 2.1 as long as combined lengths of remaining articles.

*Mandible palp* article 2 with 2 long biserrate setae, article 3 with 7 long pectinate setae (serrations not seen due to mount conditions). Right incisor with symmetrical cusps, margins convex, distally acute; left mandible incisor similar to right incisor. *Molar process* entirely smooth. Right mandible spine row composed of 10 spines; not divided by truncate lobe, without lacinoid spine. *Maxilla 1* lateral lobe with 12 strongly serrate RS, and 1 simple RS; mesial lobe with 3 long, simple RS. *Maxilla 2* lateral lobe with 4 long, curved, finely serrate setae; middle lobe with 3 long serrate setae, mesial lobe with 3 long simple setae and many long setules. *Maxilliped endite* 2.2 as long as greatest width, extending to distal margin of palp article 4, distal margin subtruncate, smooth, with shallow distomesial concavity, with 4 mesial tubercular RS, distomesial margin with 3 coupling setae. *Maxilliped palp* article 2 2.3 as long as article 1, mesial lobe extending to article 4, distomesial margin with 1 simple seta; article 3 0.6 as long as article 2, distomesial margin with 2 simple setae; article 4 3.1 as long as wide, mesial margin straight, distally with 3 setae; article 5 0.2 as long as 4, with 6 terminal setae. *Epipod* 3.8 as long as basal width, distally narrowly rounded; as long as palp, 0.4 as long as endite.

*Pereopod 1* basis 3 as long as wide, superior margin with 2 simple setae; ischium 0.6 as long as basis, 2.6 as long as wide; merus 0.7 length of ischium, 2.1 as long as wide; carpus 1.2 as long as ischium, 4.8 as long as wide; propodus 5.2 as long as wide, superior margin 2 simple setae; inferior margin with 2 acute RS, dactylus 0.4 as long as propodus, with 2 claws (and acute robust seta). Pereopods 2–7 sub-similar, more slender than pereopod 1, each with 2 claws (and acute robust seta). *Pereopod 7* basis 3.1 as long as wide; superior margin with 4 short simple setae; ischium 0.7 as long as basis, 2.8 as long as wide, superior margin weakly convex at midpoint, superior margin with 2 simple setae, inferior distal angle with 2 setae; merus 0.7 as long as ischium, 2.5 as long as wide, superodistal angle with 1 simple seta; carpus 1.1 as long as ischium, 4.9 as long as wide, inferior margin with 2 setae, superior distal angle with 1 prominent pappose seta (and 2 simple setae); propodus 1.2 as long as ischium, 5.1 as long as wide, inferior margin with 2 acute RS, superior margin with 2 simple setae (and distal penicillate seta); dactylus 0.4 as long as propodus.

*Pleopod 2* 1.2 as long as proximal width, lateral margins weakly convex, posterior margins weakly concave (with long cuticular scale-setae), apex with 2 sub-apical simple setae. *Pleopod 3* endopod 2.2 midwidth (partly fused to protopod); exopod article 1 3.6 as long as wide, extending to endopod apex, lateral margin weakly fringed with cuticular scale-setae; article 2 0.4 as long as article 1, lateral and mesial margins with short cuticular scale-setae.

*Uropod* peduncle extending to margin of pleotelson, mediodistal corner weakly produced and acute, distolateral margin 4 simple submarginal setae, mesial margin smooth. Exopod 0.7 as wide as endopod, 1.0 as long as wide, with 8 simple setae. Endopod 0.8 as long as wide, 0.3 as long as peduncle proximolateral margin, apex with 7 long simple setae.

**Male.** Not known.

#### Colour pattern.

White with narrow reticulate transverse brown band on posterior of head; interocular head band occupies 37% of head length.

#### Variation.

Pleotelson marginal spines from 5 to 7 per margin.

#### Remarks.

*Joeropsis tropida* sp. n. is the only known carinate species on the Great Barrier Reef, and the only carinate species from coral reef habitats. Undescribed carinate species are present at Ningaloo Reef, Western Australia and also at Rodrigues Island, Mauritius (personal observation).

*Joeropsis bicarinata* Just, 2001 is similar, but lacks the brown head band, has far more strongly developed dorsal carinae that also extend onto the pleotelson, is far larger (to 5.5 mm) and occurs on the shelf and slope of southeastern Australia at depths from 102 to 400 metres.

Species of *Joeropsis* usually show two types of dactylar morphology, that is all pereopods with two dactylar claws or pereopod 1 with two dactylar claws and pereopods 2–7 with three dactylar claws. *Joeropsis tropida* has all pereopods with two dactylar claws, but also has all pereopods with a dactylar robust seta in what would otherwise be the position of the third dactylar claw. This character is unique among those species for which the dactylus morphology has been recorded.

**Figure 23.**
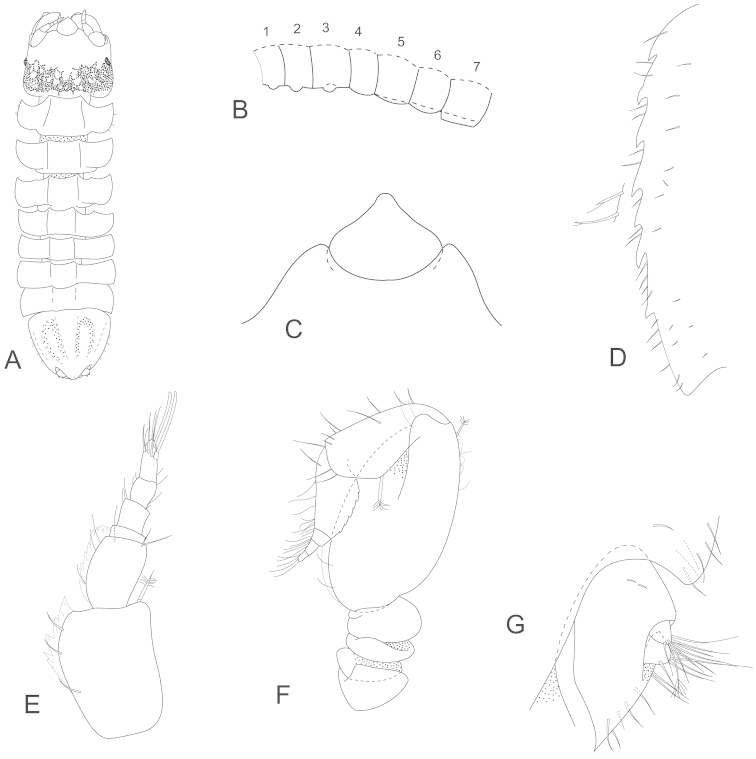
*Joeropsis tropida* sp. n. **A, B** holotype; remainder female paratype MTQ W33714. **A** dorsal view **B** sternal keels (sternites numbered) **C** pseudorostrum **D** pleotelson lateral margin **E** antenna 1 **F** antenna 2 **G** uropod.

**Figure 24.**
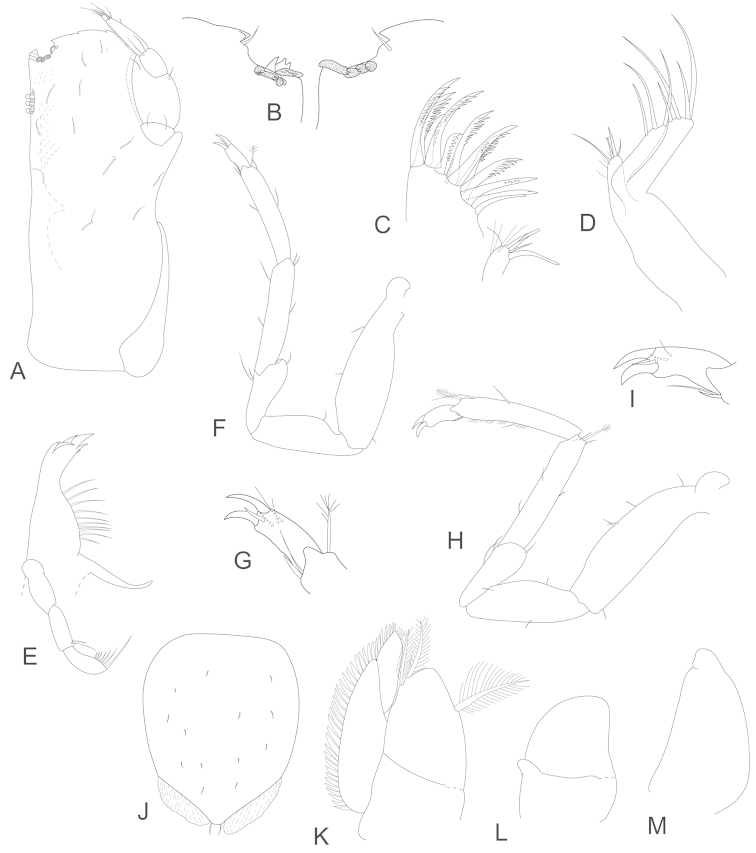
*Joeropsis tropida* sp. n. All female paratype MTQ W33714. **A** maxilliped **B** distomesial angles, maxilliped endites **C** maxillula **D** maxilla **E** left mandible **F** pereopod 7 **G** pereopod 7 dactylus **H** pereopod 1 **I** pereopod 1 dactylus **J–M** pleopods 2–5 respectively.

#### Distribution.

Known only from the type locality, Hicks Reef (Fig. 1).

#### Etymology.

From the Latin *tropidos* meaning keeled, alluding to the keeled dorsum of this species.

### Joeropsis varanus sp. n.

Taxon classificationAnimaliaIsopodaJoeropsididae

http://zoobank.org/00007DE5-065F-48C6-A702-545F2FA2DA8C

Figs 25 , 26 , 27

#### Material.

*Holotype*. ♂ (3.0 mm), North Point, Lizard Island, 14.64553°S, 145.45335°E, 12 April 2008, compacted dead *Acropora*, 0.5 m, stn CGLI-20C, coll. NLB (MTQ W31921).

*Paratypes*. ♂ (2.0 mm; dissected, two slides), Seabird Islet, Lizard Island, 14.69497°S, 145.4657°E, 23 February 2009, outer reef front, dead coral heads, 6–8 m, stn LIZ09-19B, coll. MB-P & NLB (MTQ W31923). Juv. (1.3 mm), same data as holotype (MTQ W13976). Juv. (1.8 mm), same data as holotype (MTQ W31922).

#### Description.

*Body* 3.1 as long as greatest width, dorsal surfaces smooth, without setae. *Cephalon* length 0.6 width, lateral margins evenly weakly convex, strongly serrate (anterior 4 teeth prominent). *Pseudorostrum* 0.6 as long as proximal width, anterior margin acute. *Eyes* sublateral, with ~18 ommatidia, colour dark brown. *Pereonites* compact, close to each other, without dorsal carinae; *tergite lateral margin* subtruncate, lateral margins smooth; median keels not observed. *Pleotelson* width 1.3 length; dorsal surface with single median and paired submedian low ridges, caudomedial lobe narrowly rounded; lateral margins convex, each with 8 spines (prominent).

*Antenna 1* with 5 articles; article 1 1.2 as long as wide, distolateral angle not lobed, with serrations, distomesial margin not serrate; article 2 0.5 as long as article 1, 1.3 as long as wide; lateral margins of articles 1 and 2 without cuticular scales; article 3 0.6 as long as article 2; article 4 0.7 as long as article 3; article 5 1.2 as long as article 3, 2.0 as long as proximal width, distally with 2 aesthetascs (and 2 simple setae). *Antenna 2* peduncle article 5 1.3 as long as articles 1–4 combined, 4.3 as long as article 3, 1.8 as long as wide, lateral margin weakly convex, distally concave, with small cuticular scales, mesial margin weakly convex; article 6 1.2 as long as width, distally weakly expanded, distal width 1.8 proximal width, 0.5 as long as article 5, lateral margin without cuticular scales, mesial margin with 2 simple setae, distodorsal surface without setae; flagellum with 5 articles, article 1 0.8 as long as peduncle article 6, 1.2 as long as combined lengths of remaining articles.

*Mandible palp* article 2 not observed. Right incisor with symmetrical cusps, margins convex, distally acute; left mandible incisor similar to right incisor. *Molar process* entirely smooth. Right mandible spine row composed of 8 spines; left mandible spine row composed of 9 spines, not divided by truncate lobe; without lacinoid spine. *Maxilla 1* lateral lobe with 12 strongly serrate RS, and 2 simple RS; mesial lobe with 3 long, simple RS. *Maxilla 2* lateral lobe with 4 long, curved, finely serrate setae; middle lobe with 4 long serrate setae, mesial lobe with 1 serrate and 3 long simple setae and many long setules. *Maxilliped endite* 2.1 as long as greatest width, extending to middle of palp article 4, distal margin evenly rounded, finely serrate (laterally), with shallow distomesial concavity, with 3 mesial tubercular RS (plus 1 triangular RS and one serrate RS at distomesial angle), distomesial margin with 4 coupling setae. *Maxilliped palp* article 2 2.6 as long as article 1, mesial lobe extending to mid-margin of article 3, distomesial margin with 3 simple setae; article 3 0.5 as long as article 2, distomesial margin with 1 simple seta; article 4 4.2 as long as wide, mesial margin straight, distally with 3 setae; article 5 0.2 as long as 4, with 5 terminal setae.

*Pereopod 1* basis 3.2 as long as wide, superior margin with 1 simple seta; ischium 0.8 as long as basis, 3.1 as long as wide (superior margin with 3 stiff setae); merus 0.5 length of ischium, 1.8 as long as wide; carpus 1.1 as long as ischium, 5 as long as wide (inferior margin with 3 long stiff setae); propodus 5.2 as long as wide, superior margin 3 simple setae (and prominent penicillate seta at distal angle); inferior margin with 3 acute RS (distal three-quarters with cuticular scale-setae), dactylus 0.4 as long as propodus, with 2 claws. Pereopods 2–7 sub-similar, more slender than pereopod 1, each with 3 claws. *Pereopod 7* basis 3.7 as long as wide; superior margin with 1 short simple seta, inferior margin with 3 simple setae; ischium 0.7 as long as basis, 3 as long as wide, superior margin weakly convex at midpoint, with 2 simple setae, inferior distal margin with 1 seta; merus 0.7 as long as ischium, 1.7 as long as wide, superodistal angle with 2 simple setae, inferior margin with 2 simple setae; carpus 1.1 as long as ischium, 4.4 as long as wide, inferior margin with 5 simple setae, superior margin with 1 simples seta, distal angle with 1 prominent pappose seta; propodus 1.1 as long as ischium (1.13), 5.8 as long as wide, inferior margin with 3 acute RS, superior margin with 4 simple setae; dactylus 0.5 as long as propodus.

*Pleopod 1* 1.9 as long as greatest width, lateral margin weakly concave, apical lobe narrowly rounded with strongly oblique mesial margin, with long marginal setae, lateral margin with slender setae, distolateral lobe acute, not extending to distal margin. *Pleopod 2* protopod 2.7 as long as midwidth, lateral margin mid-half weakly convex, without setae, distal margin weakly concave, with long marginal scales, apex narrowly rounded; *stylet* in retracted position reaching apex. *Pleopod 3* endopod 2.6 midwidth; exopod article 1 4.4 as long as wide, extending beyond apex, lateral margin densely fringed with cuticular scale-setae; article 2 0.4 as long as article 1, lateral and mesial margins with long cuticular scale-setae (lateral only).

*Uropod* peduncle extending slightly beyond margin of pleotelson, mediodistal corner strongly produced and acute, distolateral margin with 2 simple submarginal setae, mesial margin serrate. Exopod 0.5 as wide as endopod, 1.2 as long as wide, with 5 simple setae. Endopod 0.9 as long as wide, 0.3 as long as peduncle proximolateral margin, apex with 8 long simple setae.

**Female.** No females present in material.

#### Colour pattern.

Head with narrow, brown band running between the eyes and to lateral margin, occupying 30% of head length. Pereonites and pleotelson clear in holotype, in smaller male paratype tergite lateral margin of pereonite 2 with chromatophores; in the juvenile (MTQ W31922) tergite lateral margin of pereonites 2, 4, 6, 7 and anterior of pleotelson with chromatophores.

#### Size.

Adult males 2.0–3.0 mm.

#### Remarks.

*Joeropsis varanus* sp. n. may be identified by the short interocular head band, lateral margins of the head with strong serrations, pleotelson lateral margin with eight serrations and an acute pseudorostrum. The pereopods of *Joeropsis varanus* are more slender than in many other species.

**Figure 25.**
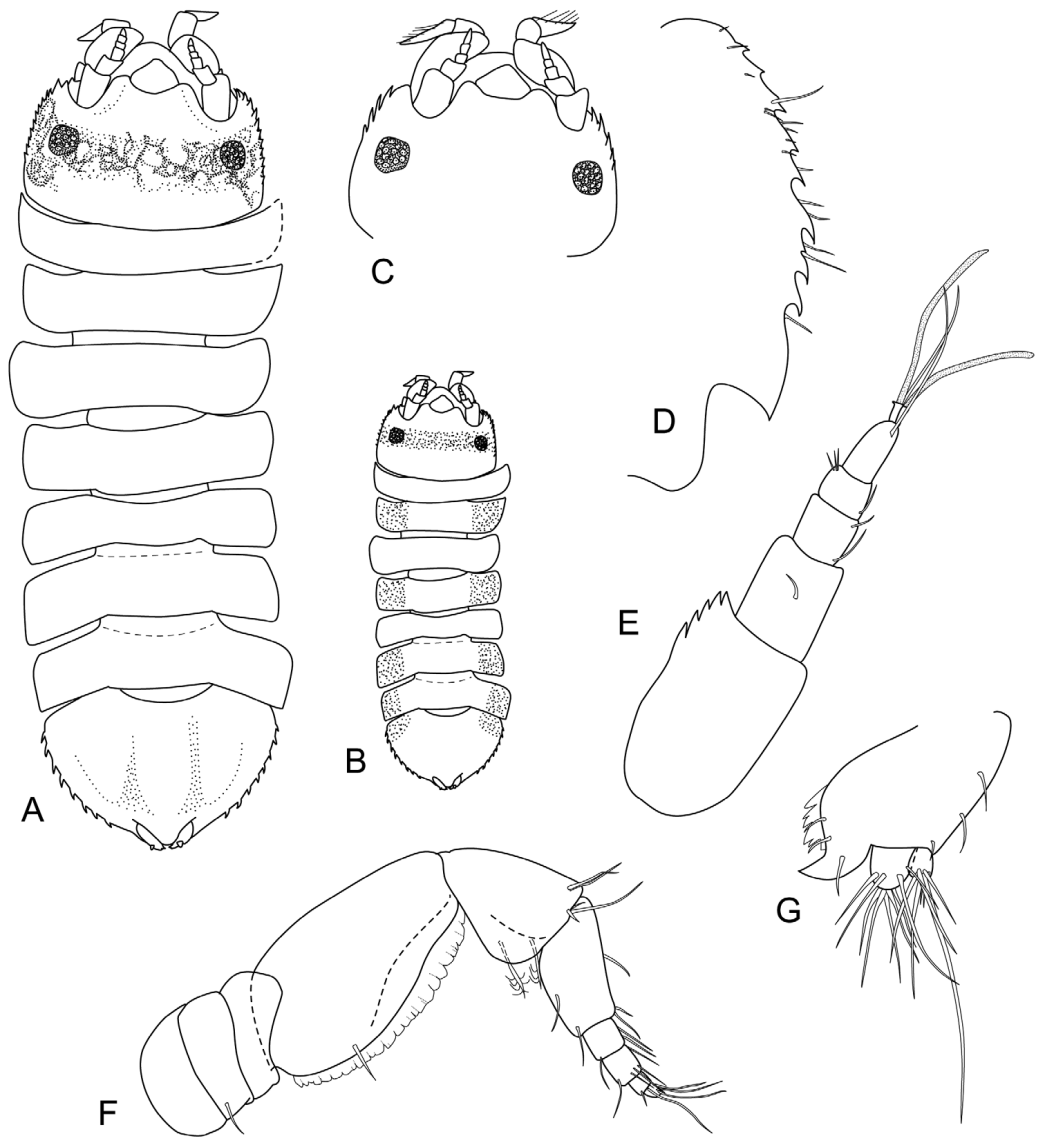
*Joeropsis varanus* sp. n. **A, C** holotype **A** dorsal view **B** dorsal view, immature (1.8 mm), coxal pattern, MTQ W31922; **C** head, dorsal view **D–G** male paratype (2.0 mm) MTQ W31923 **D** pleotelson lateral margin **E** antenna 1 **F** antenna 2 **G** uropod.

**Figure 26.**
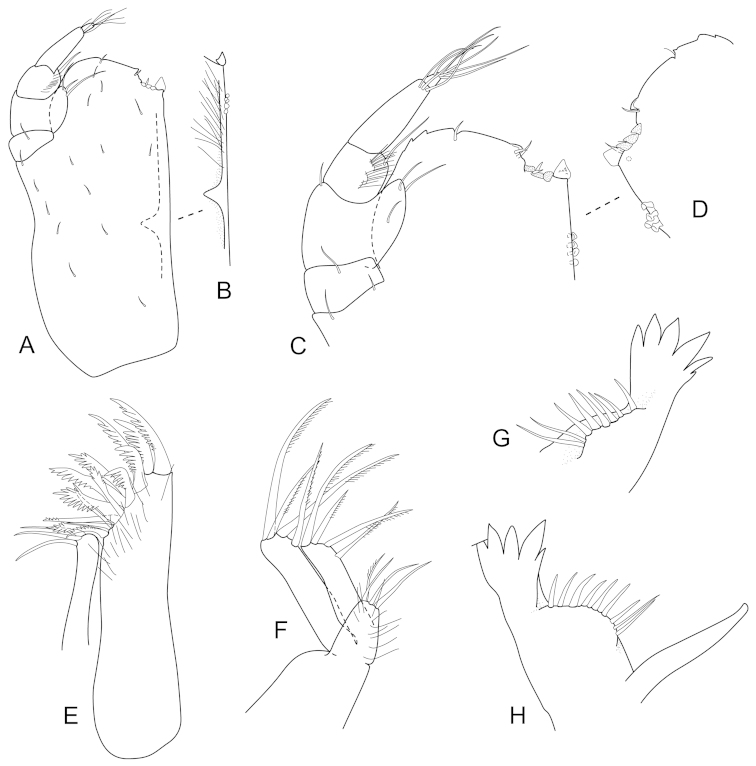
*Joeropsis varanus* sp. n. All male paratype (2.0 mm) MTQ W31923. **A** maxilliped **B** maxilliped, superomesial margin **C** right maxilliped **D** left maxilliped, distomesial margin **E** maxillula **F** maxilla **G** right mandible **H** left mandible.

**Figure 27.**
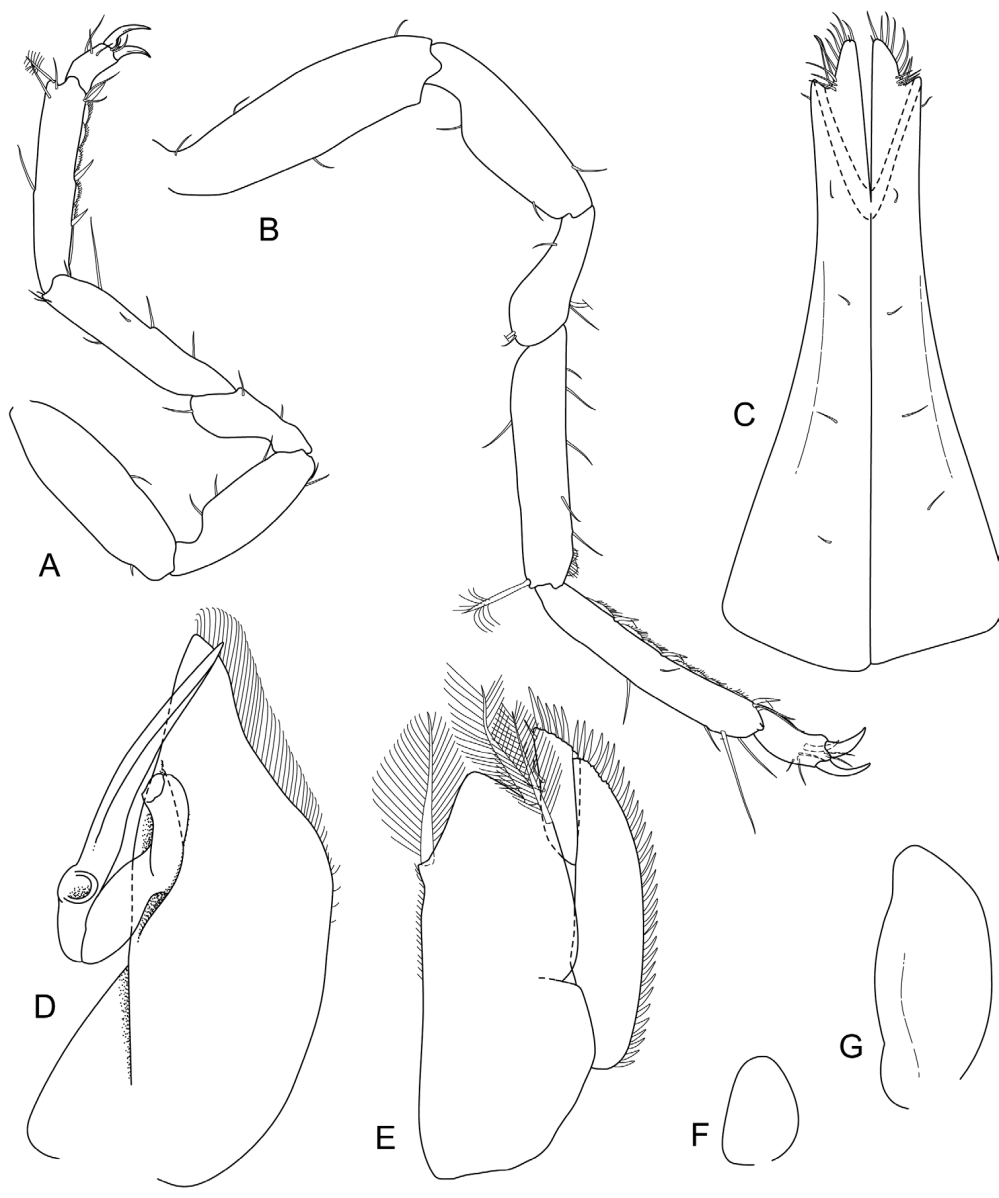
*Joeropsis varanus* sp. n. All male paratype (2.0 mm) MTQ W31923. **A** pereopod 1 **B** pereopod 7 **C–E** pleopods 1–3 respectively **F** pleopod 5 **G** pleopod 4.

#### Distribution.

Fringing reef at North Point and Seabird Islet, Lizard Island (Fig. 1); intertidal to 8 metres.

#### Etymology.

The epithet is the genus name for the Sand Monitor, *Varanus gouldi*, from which Lizard Island got its English name.

### Joeropsis wattora sp. n.

Taxon classificationAnimaliaIsopodaJoeropsididae

http://zoobank.org/096B65CB-3A76-48BA-98F3-4EF77FD55511

Figs 28 , 29 , 30

#### Material.

*Holotype*. ♂ (2.2 mm), Lizard Island, 14.6867°S, 145.4551°E, 30 August 2010, 3 m, shallow lagoon, LI10-037, coll. I. Marin (MTQ W34019).

*Paratypes*. ♂ (1.2 mm), off Palfrey Is, 14.68687°S, 145.43764°E, 16 April 2008, 7 m, hard, current swept bottom, CGLI-035, coll. MB-P (MTQ W13978). ♂ (1.6, imm 1.5 mm), ♀ (non-ovig. 2.2 [part dissected], 1.7 [dissected], 1.7, 1.6, 1.5 mm), same data as holotype (MTQ W32678).

#### Description.

*Body* 4.6 as long as greatest width, dorsal surfaces finely granular, without setae. *Cephalon* length 0.8 width, lateral margins straight, smooth. *Pseudorostrum* 0.7 as long as proximal width, anterior margin narrowly truncate. *Eyes* sublateral, with 8–12 ommatidia, colour black. *Pereonites* not compact, widely spaced, without dorsal carinae; *tergite lateral margin* subtruncate, lateral margins smooth; median keels on sternites 5–7, keels well developed. *Pleotelson* width 1.1 length, dorsal surface with single median and paired submedian low ridges, caudomedial lobe broadly rounded; lateral margins weakly convex, each with 5 spines.

*Antenna 1* with 6 articles; article 1 1.5 as long as wide, distolateral angle strongly lobed, strongly serrated, distomesial margin with single serration; article 2 0.6 as long as article 1, 1.4 as long as wide; lateral margins of articles 1 and 2 without cuticular scales; article 3 0.5 as long as article 2; article 4 0.9 as long as article 3; article 5 1.5 as long as article 3, 3.09 as long as proximal width, distally with 2 aesthetascs. *Antenna 2* peduncle article 5 1.8 as long as articles 1–4 combined, 3.7 as long as article 3, 2.6 as long as wide, lateral margin weakly convex, with small cuticular scales, mesial margin straight; article 6 1.4 as long as width, distally weakly expanded, distal width 1.9 proximal width, 0.6 as long as article 5, lateral margin without cuticular scales, mesial margin with 6 simple setae, distodorsal surface with scattered simple setae; flagellum with 6 articles, article 1 0.8 as long as peduncle article 6, 2 as long as combined lengths of remaining articles.

*Mandible palp* both damaged in dissections; right incisor with symmetrical cusps, margins convex, distally acute; left mandible incisor similar to right incisor. *Molar process* distal third finely serrate. Right mandible spine row composed of 8 spines; left mandible spine row composed of 9 spines, left mandible spine row divided by truncate lobe; without lacinoid spine. *Maxilla 1* lateral lobe with 10 strongly serrate RS, and 3 simple RS; mesial lobe with 4 long, simple RS. *Maxilla 2* lateral lobe with 4 long, curved, finely serrate setae (2 short, 2 long); middle lobe with 4 long serrate setae (2 short, 2 long), mesial lobe with 4 long simple setae and many long setules. *Maxilliped endite* 2.2 as long as greatest width, extending to middle of palp article 4, distal margin subtruncate, finely serrate, with shallow distomesial concavity, with 4 mesial tubercular RS, distomesial margin with 3 coupling setae. *Maxilliped palp* article 2 1.4 as long as article 1, mesial lobe extending to mid-margin of article 3, distomesial margin with 2 simple setae; article 3 0.7 as long as article 2, distomesial margin with 1 simple seta; article 4 5.4 as long as wide, mesial margin weakly concave, distally with 4 setae; article 5 0.2 as long as 4, with 8 terminal setae. *Epipod* 3.6 as long as basal width, distally acute; 1.1 as long as palp, 1.9 as long as endite.

*Pereopod 1* basis 3.4 as long as wide, superior margin with 3 simple setae, inferior margin with 3 simple setae; ischium 0.7 as long as basis, 3.3 as long as wide superior margin with 1 simple seta, inferior margin with 2 simple setae; merus 0.6 length of ischium, 1.9 as long as wide superior margin with 1 simple seta, inferior margin with 1 simple seta and 1 submarginal simple seta; carpus 1.1 as long as ischium, 3.95 as long as wide, superior margin with 1 short simple seta, inferior margin with 4 simple seta; propodus 5.3 as long as wide, superior margin 2 simple setae (1 brush-tipped; distal angle only); inferior margin with 2 acute RS, dactylus 0.4 as long as propodus, with 2 claws. Pereopods 2–7 sub-similar, more slender than pereopod 1, each with 3 claws. *Pereopod 7* basis 3.4 as long as wide; superior margin with 5 short simple setae; ischium 0.8 as long as basis, 3.4 as long as wide, superior margin weakly convex at midpoint, superior margin with 2 simple setae, inferior distal angle with 0 setae (inferior margin with 2 setae); merus 0.6 as long as ischium, 1.9 as long as wide, superodistal angle with 1 simple seta (weak cuticular scales), inferior margin with 3 simple seta; carpus 0.9 as long as ischium, 4.8 as long as wide, inferior margin with 4 setae, superior distal angle with 3 simpee setae; propodus 1.1 as long as ischium, 6.3 as long as wide, inferior margin with 2 acute RS, superior margin with 3 simple setae and prominent pappose seta; dactylus 0.4 as long as propodus.

*Pleopod 1* 2.4 as long as greatest width, lateral margin strongly concave, apical lobe narrowly rounded, mesial margin weakly oblique, with long marginal setae, lateral margin with slender setae, distolateral lobe acute, not extending to distal margin. *Pleopod 2* protopod 2.6 as long as midwidth, lateral margin mid-half weakly convex, without setae, distal margin weakly concave, with long marginal cuticular scales, apex narrowly rounded; *stylet* in retracted position extending beyond apex.

*Uropod* peduncle extending slightly beyond margin of pleotelson, mediodistal corner weakly produced and acute, distolateral margin 2 simple submarginal setae, mesial margin smooth. Exopod 0.8 as wide as endopod, 1.4 as long as wide, with 8 simple setae. Endopod 1.3 as long as wide, 0.4 as long as peduncle proximolateral margin, apex with 6 long simple setae.

**Female.** Female dissection of pleopods failed.

#### Size.

Males 1.3–2.2 mm, mean 1.4 mm (*n*=3); non-ovigerous females 1.5–2.1 mm, mean 2.7 mm (*n*=5); all from the type series.

#### Remarks.

The colour pattern of *Joeropsis wattora* sp. n. is closely similar to species such as *Joeropsis makrogenys* sp. n. and *Joeropsis sandybrucei*, both with a wide, dark brown head band. *Joeropsis wattora* can be identified by the elongate body (4.7 as long as wide) with widely spaced pereonites, anteriorly narrowed pseudorostrum (the pseudorostrum is anteriorly concave in both *Joeropsis makrogenys* sp. n. and *Joeropsis sandybrucei*) and the small size (mean adult length 1.6 mm).

**Figure 28.**
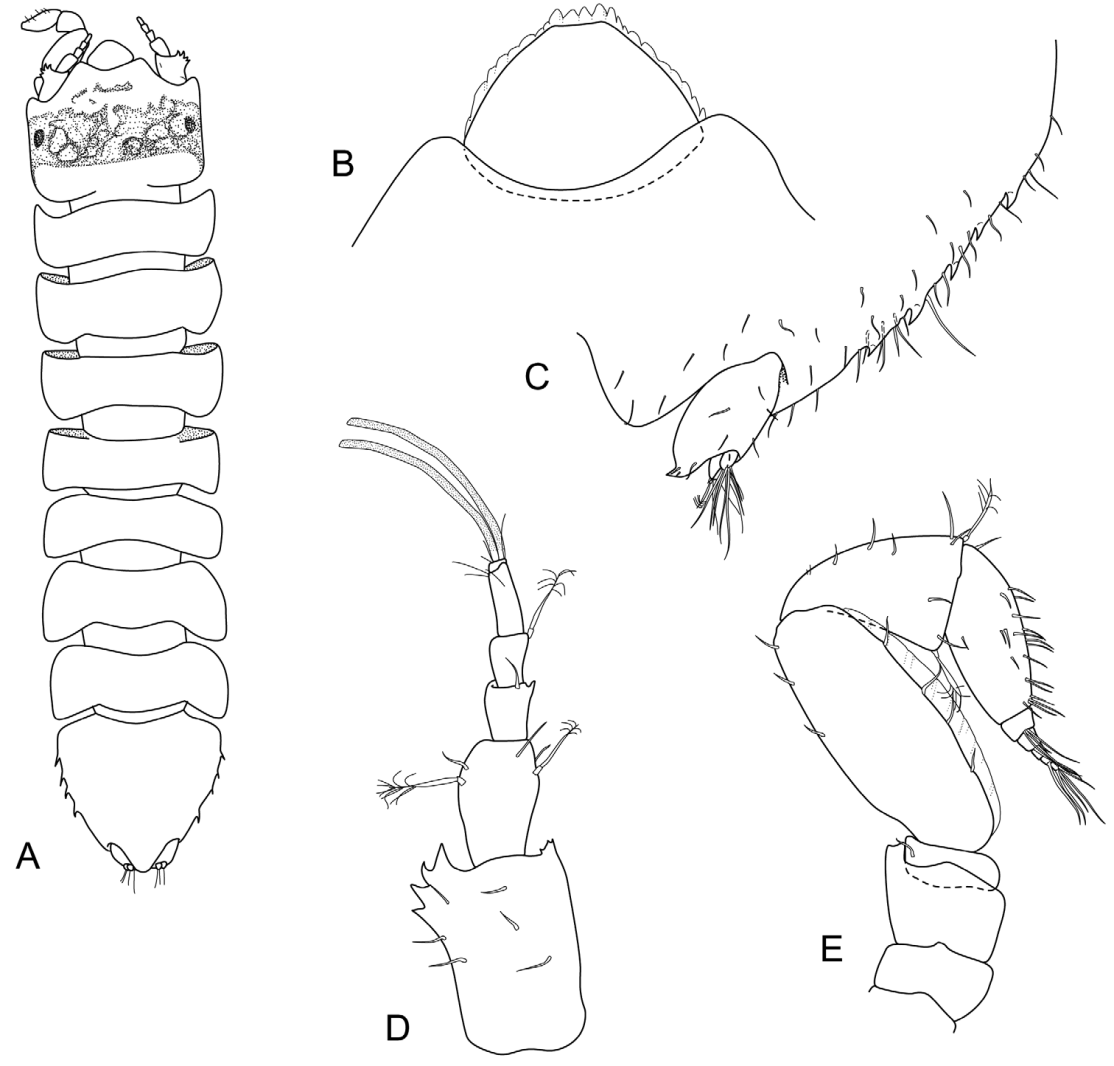
*Joeropsis wattora* sp. n. **A, B** holotype; **C** paratype (2.2 mm), **D, E** female paratype (1.7 mm) MTQ W32678. **A** dorsal view **B** pseudorostrum **C** pleotelson lateral margin **D** antenna 1 **E** antenna 2.

**Figure 29.**
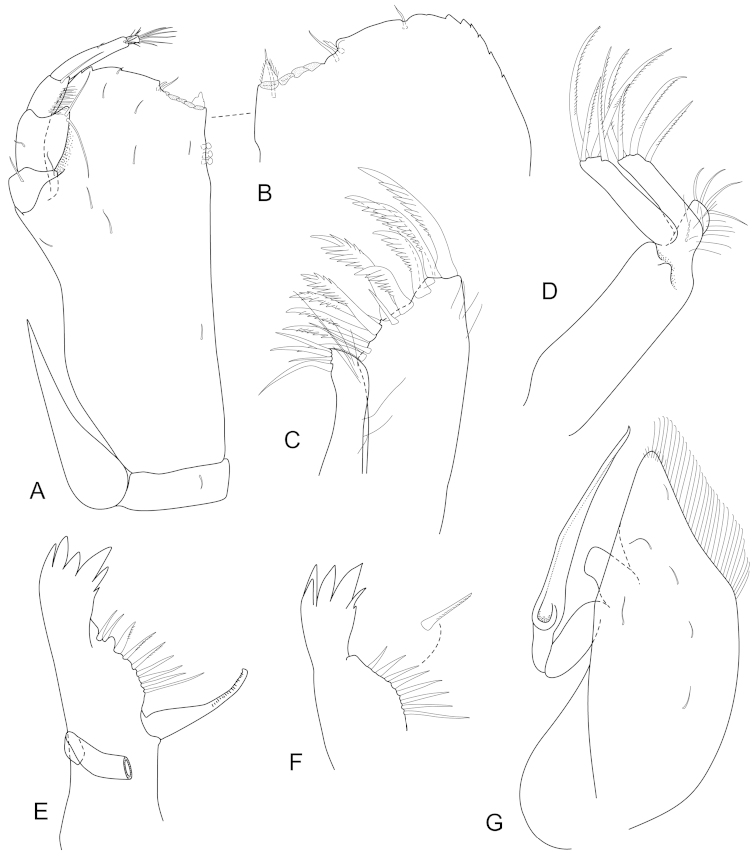
*Joeropsis wattora* sp. n. Female paratype (1.7 mm) MTQ W32678. **A** maxilliped **B** maxilliped endite, distomesial margin **C** maxillula **D** maxilla **E** left mandible **F** right mandible **G** pleopod 2.

**Figure 30.**
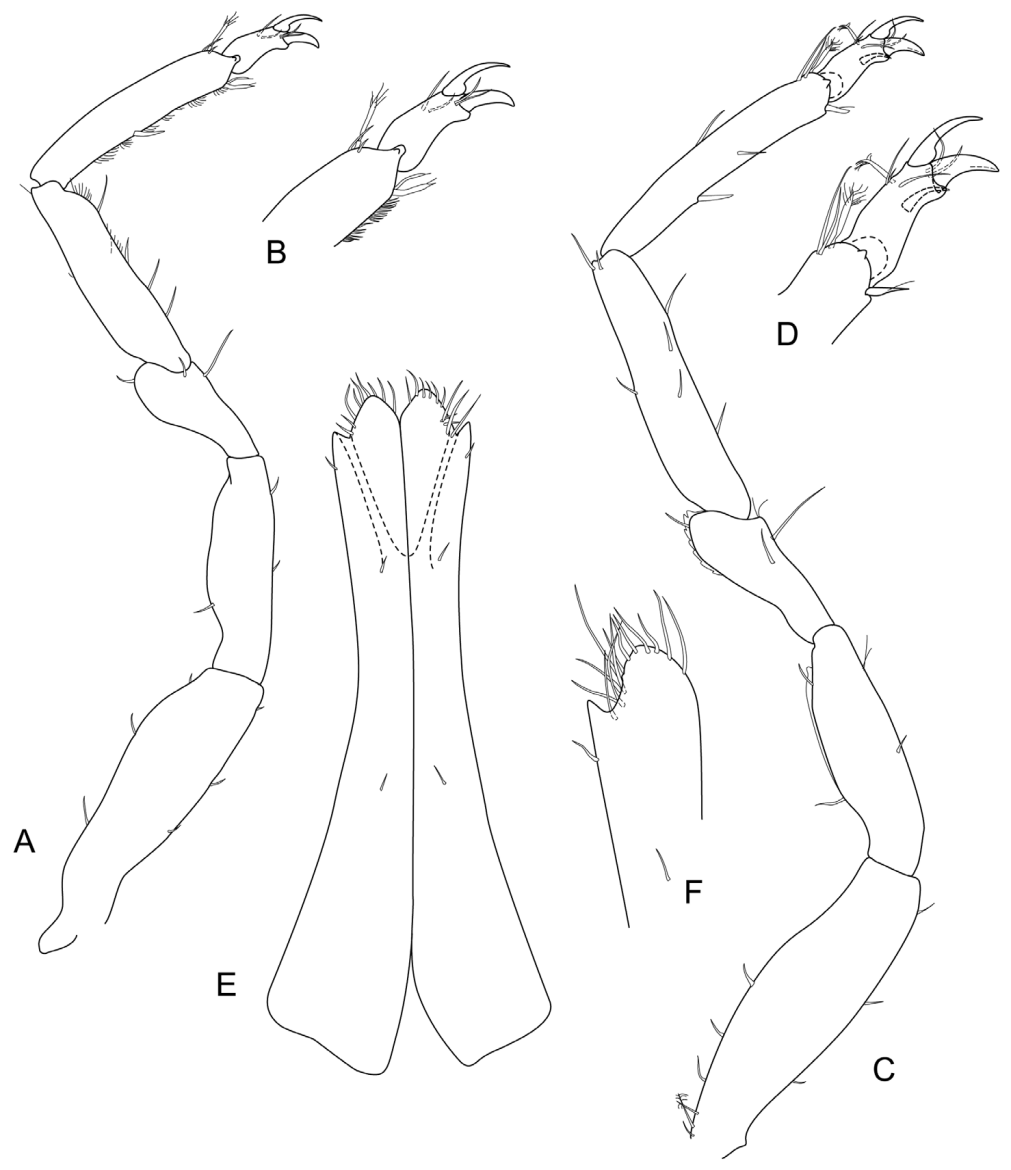
*Joeropsis wattora* sp. n. **A–D** female paratype (2.2 mm) MTQ W32678 **E, F** holotype. **A** pereopod 1 **B** pereopod 1 dactylus **C** pereopod 7 **D** pereopod 7 dactylus **E** pleopod 1 pleopod 1 apex.

#### Variation.

Pleotelson spines range from 3 to 5 per margin, with large specimens having 3 or 4 spines.

#### Distribution.

Lizard Island lagoon and off Palfrey Island, Lizard Island Group (Fig. 1); 3–7 metres.

#### Etymology.

The epithet wattora is an Aboriginal word meaning long, in the sense of elongate; noun in apposition.

### Species not described

The following species lacked adequate material for description. In most cases the principal differentiating characters from other species in the region (and elsewhere) are a combination of size, colour pattern, setosity and shape of the pseudorostrum.

#### Joeropsis sp. 9

Taxon classificationAnimaliaIsopodaJoeropsididae

##### Material.

(1.9 mm), Hicks Reef, 14.44803°S, 145.4992°E, 21 February 2009, outer reef front, dead coral heads on bommies, 5–6 m, stn LIZ09-16H, coll. NLB & MB-P (MTQ W34020).

##### Remarks.

The colour pattern of a short head band, and short band on pereonite 4 with very few chromatophores laterally is unique; the pseudorostrum is laterally rounded and anteromedially weakly concave, the eyes are small and marginal and the pleotelson margins have 4 and 5 spines on each margin (counted under compound microscope). The single specimen lacks both antenna and pereopod 1. Despite the apparently unique colour pattern the species cannot be described without further material.

#### Joeropsis sp. 11

Taxon classificationAnimaliaIsopodaJoeropsididae

##### Material.

♂ (1.5 mm), Hicks Reef, 14.44803°S, 145.4992°E, 21 February 2009, outer reef front, dead coral heads on reef edge, 5–7 m, LIZ09-16E, coll. NLB & MB-P (MTQ W34021). ♀ (non-ovig. 1.4 mm), Day Reef, 14.48356°S, 145.5459°E, 13 February 2009, outer reef, fine rubble, 10 m, stn LIZ09-04C, coll. MB-P (MTQ W31200). ♀ (1.2 mm), Day Reef, 14.48283°S, 145.5564°E, 19 February 2009, outer reef front, small rubble in gully, 7.5 m, stn LIZ09-13A, coll. MB-P & N. Bruce (MTQ W34022).

##### Remarks.

No specimen has antenna. The dorsum is finely and sparsely speckled with black chromatophores, with further speckling ventrally. The pseudorostrum is laterally angled and appears to have marginal cuticular scales.

#### Joeropsis sp. 12

Taxon classificationAnimaliaIsopodaJoeropsididae

##### Material.

♀ (non-ovig. 3.2 mm), Hicks Reef, 14.44803°S, 145.4992°E, 21 February 2009, outer reef front, dead coral heads on reef edge, 5–7 m, LIZ09-16E, coll. NLB & MB-P (MTQ W31288).

##### Remarks.

The single specimen lacks both antenna. The dorsum is more setose than in other species and is covered in pale-brown chromatophores, these being more dense on the head forming a short (40% head length) band; the pseudorostrum is evenly rounded, the uropods have a prominent terminal spine and the pleotelson lateral margins each have 6 spines. There are no other similar species in the region; *Joeropsis adusta* sp. n. is smaller, less densely setose, and appears evenly dark brown; *Joeropsis salvati* Müller, 1989 is smaller still, and has a slightly truncate pseudorostrum.

#### Joeropsis sp. 15

Taxon classificationAnimaliaIsopodaJoeropsididae

##### Material.

♂ (3.0 mm), Day Reef, 14.47045°S, 145.52840°E, 5 September 2010, outer reef, dead corals, 17 m, LI10-077B, coll. CB (MTQ W32849). ♀ (non-ovig. 2.0 mm), Day Reef, 14.47045°S, 145.52840°E, 5 September 2010, 17 m, outer reef, dead coral heads, LI10-077B, coll. CB (MTQ 34023).

##### Remarks.

A relatively large species, with a prominent, posteriorly acute keel on sternite 7 that extends posteriorly over pleopods in both the male and female specimen. The male has a short diffuse head band, and weak mottling on the tergite lateral margin of pereonites 2, 5 and 7, laterally on pereonite 7 and very lightly at anterolateral angles of the pleotelson. The female pleopod 2 differs from other species in having a distinct subterminal inflection to the lateral margins. The pseudorostrum is anteriorly concave. The male specimen lacks antenna.

#### Joeropsis sp. 16

Taxon classificationAnimaliaIsopodaJoeropsididae

##### Material.

3♂ (2.1, 1.7, 1.5 mm), Bommie Bay, Lizard Island, 14.66157°S, 145.47160°W, 8 September 2010, dead coral, 8 m, stn LI10-101B, coll. CB (MTQ W34024).

##### Remarks.

These specimens are similar to *Joeropsis adusta* sp. n. differing primarily in having a sub-quadrate pseudorostrum that has a weakly convex anterior margin and being reddish–brown rather than dark brown. *Joeropsis lentigo* Kensley & Schotte, 2002, from the Seychelles, is also similar, differing in having the anterior margin of the pseudorostrum more strongly convex. The latter species is not described in sufficient detail to make further comparisons.

## Supplementary Material

XML Treatment for Joeropsididae

XML Treatment for Joeropsis

XML Treatment for Joeropsis adusta

XML Treatment for Joeropsis goobita

XML Treatment for Joeropsis jiigurru

XML Treatment for Joeropsis makrogenys

XML Treatment for Joeropsis mije

XML Treatment for Joeropsis panstikta

XML Treatment for Joeropsis specca

XML Treatment for Joeropsis tropida

XML Treatment for Joeropsis varanus

XML Treatment for Joeropsis wattora

XML Treatment for Joeropsis sp. 9

XML Treatment for Joeropsis sp. 11

XML Treatment for Joeropsis sp. 12

XML Treatment for Joeropsis sp. 15

XML Treatment for Joeropsis sp. 16

## Footnotes

‘Bommie’ (contracted from bombora, in turn derived from the Dharuk Aboriginal word bumbora) refers to the large free–standing coral outcrops, often a massive *Porites* colony, usually extending to just below low-tide level; these structures can reach 10 metres in height. Used widely on collection data labels; Bommie Bay, Lizard Island is named for these structures.
